# Kinetic Parameters at High-Pressure-Limit for Unimolecular Alkene Elimination Reaction Class of Fatty Acid Alkyl Esters (FAAEs)

**DOI:** 10.3390/molecules30204054

**Published:** 2025-10-11

**Authors:** Xiaohui Sun, Zhenyu Pei, Zerong Li, Yuanyuan Tian

**Affiliations:** 1 School of Energy Engineering, Shanxi College of Technology, Shuozhou 036000, China; peizhenyu@sxct.edu.cn (Z.P.); tianyuanyuan@sxct.edu.cn (Y.T.); 2 College of Chemistry, Sichuan University, Chengdu 610064, China

**Keywords:** unimolecular alkene elimination reaction class, high-pressure-limit kinetic parameters, rate rules, isodesmic reaction method

## Abstract

The unimolecular alkene elimination reaction class of fatty acid alkyl esters (FAAEs) is a crucial component in the low-temperature combustion mechanism for biodiesel fuels. However, thermo-kinetic parameters for this reaction class are scarce, particularly for the large-size molecules over four carbon atoms and intricate branched-chain configurations. Thermo-kinetic parameters are essential for constructing a reaction mechanism, which can be used to clarify the chemical nature of combustion for biodiesel fuels. In this paper, the B3LYP method, in conjunction with the 6-311G(d,p) basis set, is used to carry out geometry optimization of the species participating in the reactions. Frequency calculations are further executed at the same level of theory. Additionally, coupled with the 6-311G(d,p) basis set, the B3LYP method acts as the low-level ab initio approach, while the Gaussian-4 (G4) composite method serves as the high-level ab initio approach within the isodesmic reaction correction scheme. The CCSD(T) approach is employed to verify the consistency of the electronic energy ascertained through the G4 method. The isodesmic reaction method (IRM) is used to obtain the energy barriers and reaction enthalpies for unimolecular alkene elimination reaction class of FAAEs. Based on the reaction class transition state theory (RC-TST), high-pressure-limit rate coefficients were computed, with asymmetric Eckart tunneling corrections applied across 500~2000 K temperature range. Rate rules at the high-pressure-limit are obtained through the averaging of rate coefficients from a representative collection of reactions, which incorporate substituent groups and carbon chains with different sizes and lengths. Ultimately, the energy barriers, reaction enthalpies, and rate rules at the high-pressure-limit and kinetic parameters expressed as (A, n, E) are supplied for developing the low-temperature combustion mechanism of biodiesel fuels.

## 1. Introduction

The world’s largest energy source is fossil fuels (oil, gas, and coal). Hydrocarbons and their derivatives are their primary components, with advantages of high energy content, high density, and reasonable cost. However, their use produces GHG emissions, exacerbating global warming, and climate change [1]. This emphasizes the need for alternative energy sources to meet global energy demands and reduce environmental impact. Multiple renewable energy resources, like wind, solar, hydropower, biomass, and biofuels, can substitute fossil fuels to mitigate their negative consequences. Among them, biofuels (especially biodiesel) have become prominent due to their sustainable and biodegradable features, compatibility with existing energy frameworks, and ability to reduce carbon emissions. Biodiesel, referring to fatty acid alkyl esters (FAAEs), is more viscous and dense than diesel [2,3]. Furthermore, biodiesel fuels are mainly made up of saturated and unsaturated alkyl esters with carbon chains of 12 or more atoms in length compounds derived from plant oils or animal fats via the esterification process [4,5,6]. Consequently, the recent large-scale adoption of biodiesel has motivated extensive research initiatives to acquire a systematic understanding of the chemistry underlying biodiesel combustion. For the purpose of understanding fuel combustion (a complex physical and chemical process that includes turbulence, chemical reactions, thermal radiation, and heat transfer), researchers commonly construct comprehensive combustion mechanisms to aid in grasping the combustion process of biodiesel fuels.

The comprehensive combustion mechanism of fuels is inherently complex, typically encompassing thousands of elementary reactions and involving hundreds of distinct chemical species. As the count of carbon atoms present in the fuel molecules increases, there is a corresponding and significant rise in both the numerical value of species and the intricacy of the reactions involved. When it comes to constructing comprehensive reaction mechanisms for alkanes, a systematic approach is generally employed, utilizing automatic mechanism generation programs as referenced in sources. These automatically generated mechanisms are composed of two primary sections: the first section is a reaction base, which encompasses all the reactions that involve small radicals or molecules. A common example of this is the C0~C4 reaction base, which includes reactions of species with up to four carbon atoms. The second section deals with reactions involving larger species. This part is constructed by classifying reactions into distinct reaction classes that are founded on the specific characteristics of the reaction center. A rate rule is systematically applied to each of these classes, serving to assign appropriate rate coefficients to the reactions within the respective class and ensuring the kinetics of the overall mechanism are accurately represented. This dual-component approach allows for a structured and efficient generation of reaction mechanisms, accommodating the vast complexity inherent in the combustion processes of fuels [7,8].

**Scheme 1:**
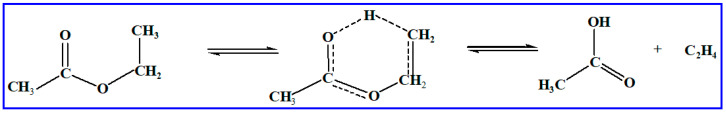
Unimolecular alkene elimination reaction of ethyl acetate with six-membered transition state: ethylene and acetic acid formation.

The few models [9,10] available for ester oxidation have been founded on the same reaction classes as alkanes. Molecular reactions are the only reaction class specific to esters. Detailed chemical kinetic mechanisms for fatty acid alkyl esters (FAAEs) often lack accurate thermo-kinetic parameters for critical unimolecular decomposition pathways, such as alkene elimination, due to the scarcity of experimental data. High-level theoretical calculations provide a powerful alternative to fill this data gap. For instance, Metcalfe et al. [9] and Schwartz et al. [11] proved that the unimolecular alkene elimination reaction (involving a six-membered transition state and producing ethylene and an acid molecule formation) holds importance for ethyl esters. The reaction processes are listed in Scheme 1. In prior work, several studies have focused on methyl butanoate. As an example, Truhlar et al. [12] investigated the kinetics of hydrogen abstraction from methyl butanoate by H atoms, while Dibble et al. [13] explored the isomerization reactions of methyl butanoate. Metcalfe et al. [9] studied the unimolecular alkene elimination for ethyl propanoate producing propanoic acid and ethylene based on the experiment. However, the rate coefficients for large-size molecules of unimolecular alkene elimination reaction for fatty acid alkyl esters (FAAEs) are scarce. High-level theoretical methods, such as the CCSD(T) and QCISD(T) [14,15] methods, are only applied to the small-size systems, and the kinetic parameters corresponding to large-size molecules are usually approximately estimated from similar reaction classes in alkanes. Therefore, a promising method should be proposed to solve this problem.

The unimolecular alkene elimination reaction is a crucial class of decomposition pathways in the reaction mechanism of biodiesel. Within this work, 55 reactions for the FAAEs unimolecular alkene elimination reaction class are chosen, with arrangement based on molecular size in ascending order. The reactions are presented in Table 1, wherein Reaction R1 is chosen as the reference reaction, and the other reactions within the class are designated as target reactions.

The objectives of this study are as follows: (1) Providing accurate energy barriers, reaction enthalpies, high-pressure-limit rate coefficients, and rate rules for unimolecular alkene elimination reaction of large-size fatty acid alkyl esters (FAAEs), which are usually calculated via analogy to alkanes. (2) Providing the quantitative and systematic influence of alkyl chain architecture—specifically its length and degree of branching—on the energy barriers and rate coefficients. This study aims to establish a comprehensive and theoretically consistent set of high-pressure-limit rate parameters for alkene elimination reaction class of FAAEs. The data presented here are intended to serve as a reliable reference for future kinetic model development and to enhance the predictive understanding of biodiesel combustion, rather than to replicate past experimental measurements. The findings from this study are expected to yield transferable insights valuable for both fundamental chemical knowledge and applied fields such as fuel design and molecular engineering.

## 2. Results and Discussion

### 2.1. Geometric Structures of the Reaction Center in Transition States

**Figure 1:**
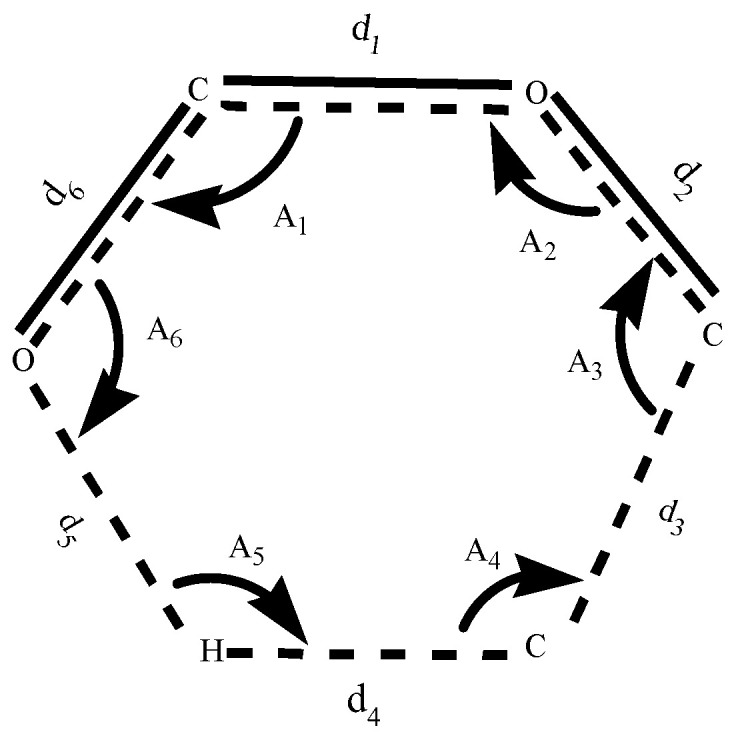
Reaction center geometries in transition states.

The reaction center associated with transition states in the reaction class under our examination is characterized by a six-membered ring. The annotation of relevant bond lengths and bond angles are depicted in Figure 1, where A_1_, A_2_, A_3_, A_4_, A_5_, and A_6_ denote bond angles, and d_1_, d_2_, d_3_, d_4_, d_5_, and d_6_ indicate bond lengths, respectively. The average values and maximum absolute deviation of the geometric parameters in the reaction center of the transition states are presented in Table 2.

Upon a meticulous scrutiny of Table 2, it is apparent that the maximum absolute deviations in the bond lengths and bond angles at the reaction centers within the geometric configuration of the transition state are 0.15 Å and 6.40°, respectively. Consequently, any target reaction, after being subtracted by the reference reaction, can be considered an isodesmic reaction. In this study, all optimized geometrical parameters associated with the reaction centers of each transition state are presented in Table S1 of the Supplementary Materials.

### 2.2. Energy Barriers

#### 2.2.1. Validation of the G4 Method in Calculating Energy Barriers

**Figure 2:**
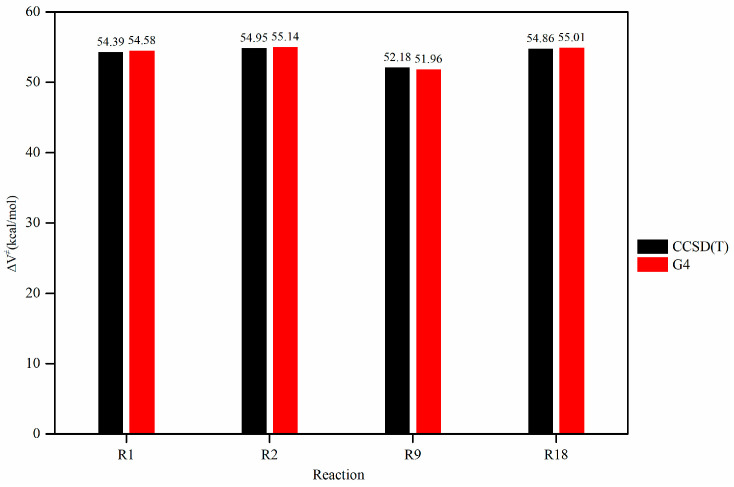
Comparison of the energy barriers between the CCSD(T)/cc-PVTZ method and G4 method.

The precision of energy barriers is crucial for ensuring the reliability of rate coefficients [16]. Consequently, the G4 composite method, which is less computationally demanding, has been selected as the high-level ab initio method for the correction scheme in isodesmic reactions. To validate the consistency of the G4 method for our specific systems, a comparative analysis with CCSD(T)/cc-pVTZ calculations has been performed in this work. A selection of four representative reactions (R1, R2, R9, and R10) from Table 1 was chosen for this purpose, with detailed findings shown in Figure 2.

As depicted in Figure 2, the energy barriers calculated by both methods show excellent agreement, with deviations of only −0.19 kcal/mol, −0.19 kcal/mol, 0.22 kcal/mol, and −0.15 kcal/mol, respectively. This close correspondence, well within the range of chemical accuracy (1~2 kcal/mol), confirms that the G4 method delivers energetics consistent with other high-level theory for these reactions.

#### 2.2.2. Assessment of the B3LYP Method and Dispersion Effects

To elucidate the effect of dispersion correction on the energy barriers associated with the unimolecular alkene elimination reactions of fatty acid alkyl esters (FAAEs), five reactions (R9, R22, R38, R49, and R55) were randomly selected from Table 1. The energy barriers for these reactions were determined using the B3LYP functional incorporating Grimme’s D3 dispersion correction (B3LYP-D3) and were contrasted with the standard B3LYP results. The comparative data are presented in Table 3.

As presented in Table 3, the disparities in energy barriers between the two methods are relatively minor, ranging from 0.49 to 1.94 kcal/mol, with a mean absolute deviation (MAD) of 1.12 kcal/mol. These values fall within the scope of chemical accuracy (1~2 kcal/mol). It indicates that the dispersion effects have only a negligible impact on the energy barriers of this reaction class. Furthermore, the B3LYP method is exclusively chosen as the low-level ab initio method within the isodesmic reaction correction scheme, then the isodesmic reaction method will be utilized to correct the energy barriers at the B3LYP level. Considering the negligible impact of dispersion corrections on the energy barriers and the procedure of the isodesmic reaction method, the B3LYP method is deemed appropriate for this work. In the following Section 2.2.4, a detailed comparison of the energy barriers before and after correction by the isodesmic reaction method at the B3LYP level with the G4 method are discussed in this work. Moreover, the explanations of the isodesmic reaction correction scheme are presented in the Section 3.

#### 2.2.3. Energy Barrier for Reference Reactions

In the present study, reaction R1 from Table 1 has been chosen as the reference reaction, with the remaining reactions in Table 1 serving as target reactions. The reaction enthalpy and energy barrier for the reference reaction are computed via the B3LYP and G4 methods. The disparities between these two methods are presented in Table 4. As indicated in Table 4, there are significant differences in the reaction enthalpy and energy barrier between the approximate method and the accurate method. The revised values for the reaction enthalpy and energy barrier are 1.69 and 6.31 kcal·mol^−1^, respectively, which will be utilized to adjust the reaction enthalpies and energy barriers derived from the low-level B3LYP method for target reactions, in accordance with Equations (6) and (7).

#### 2.2.4. Validation of the Correction Scheme for Energy Barriers in the Isodesmic Reaction Method

**Figure 3:**
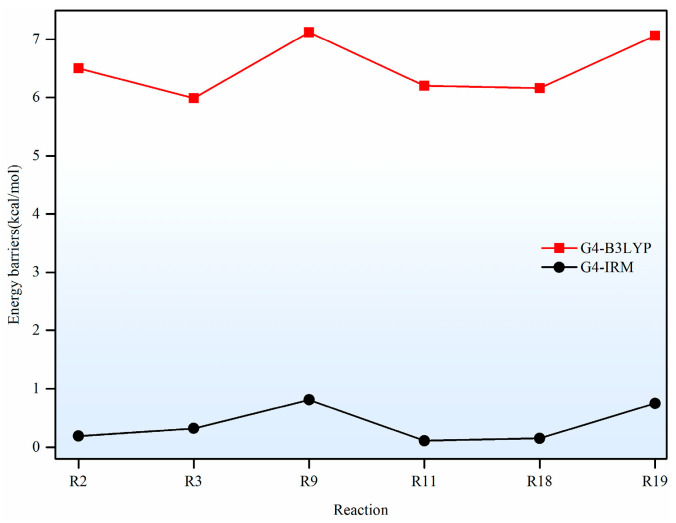
Deviations of energy barriers before and after correction via the isodesmic reaction method.

For the purpose of validating the accuracy of the energy barriers corrected by the isodesmic reaction correction scheme, six representative target reactions are chosen by contrasting the energy barriers derived from the correction scheme of the isodesmic reaction method against those computed via the G4 method and the B3LYP method. The results are presented in Table 5. In order to intuitively reflect the deviations before and after correction by the isodesmic reaction method at the B3LYP level, the absolute values of the deviations in energy barriers before and after correction are shown in Figure 3.

As presented in Table 5 and Figure 3, the disparities in energy barriers between the B3LYP method and those obtained via the G4 method range from 5.99 to 7.12 kcal·mol^−1^. These differences are substantially larger than the generally acknowledged chemical accuracy, which is typically around 1 to 2 kcal·mol^−1^. This divergence highlights the inherent limitations of B3LYP in accurately predicting energy barriers when compared with more sophisticated methods such as the G4 method. Nevertheless, a remarkable improvement is noted when the isodesmic reaction method is employed to correct these energy barriers at the B3LYP level. After applying the isodesmic reaction correction scheme, the deviations are significantly reduced to a range of −0.32 to 0.81 kcal·mol^−1^. This narrowed range falls well within the realm of chemical accuracy, thereby suggesting that the energy barriers derived from a low-level ab initio method, such as the B3LYP method, can indeed be corrected through the isodesmic reaction method. This correction scheme effectively enhances the precision of the energy barriers, enabling the attainment of values that are both dependable and accurate for practical applications in chemical research and modeling.

#### 2.2.5. Influence of the Molecular Structure on the Energy Barriers

To elucidate the structure-activity relationships, a systematic analysis is conducted on the effects of the carbon chain length and the branching degree of the alkyl groups in the esters on the energy barriers.

**Figure 4:**
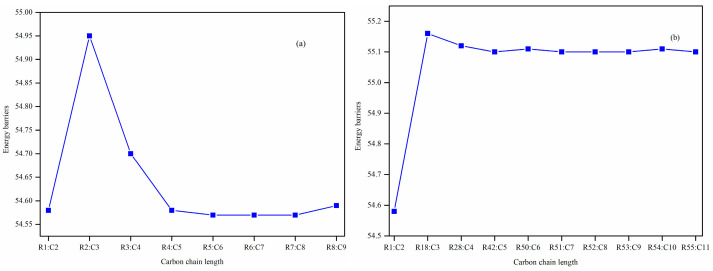
The influence of the carbon chain length on the energy barriers. (**a**) acetic acid ester series; (**b**) ethyl ester series.

Firstly, the influence of the carbon chain length on energy barriers was investigated by comparing reactions involving linear alkyl chains. In the acetic acid ester series (R1-R8), where the alcohol chain length increases from ethyl (C2) to nonyl (C9), the energy barriers remain nearly constant (approximately 54.57 kcal/mol) beyond butyl (C4), as depicted in Figure 4a. Moreover, the variation in energy barriers is less than 0.4 kcal/mol. When the acid chain is extended while the alcohol moiety is maintained as ethanol (R1, R18, R28, R42, R50–R55), the energy barriers stabilize at around 55.10 kcal/mol after butyric acid (C4) , as depicted in Figure 4b. This clear trend indicates that the six-membered ring transition state is highly localized, and the carbon chain length effectively insulates the reaction center from the distal part of the chain. The electronic and steric perturbations from remote segments of the long alkyl chains have an insignificant effect on the energy barrier, as they are too far from the reaction center.

Secondly, the energy barriers of linear and branched isomers are compared in Table 6. It is noteworthy that the substituent groups attached to the carbon directly linked to the ester oxygen are designated as the α-position, whereas the adjacent one is the β-position.

The results show that within the C3 alcohol series of acetic acid esters, the energy barrier of the α-branched isomer (R9, isopropyl) is 3.8 kcal/mol lower than that of the linear isomer (R2, n-propyl). In the C4 alcohol series of acetic acid esters, the energy barrier of the α-branched isomer (R10, sec-butyl) is 3.91 kcal/mol lower than that of the linear isomer (R3). Likewise, within the C4 alcohol series of butyric acid esters, the energy barrier of the α-branched isomer (R31) is 3.30 kcal/mol lower than that of the linear isomer (R39). This significant reduction is attributed to the stronger electron-donating inductive effect of branched alkyl groups compared to their linear isomers. The increased electron density provided by the branched group better stabilizes the transition state, thereby lowering the energy barrier. However, the energy barrier of the β-branched isomer (R11, isobutyl) is 1.01 kcal/mol higher than that of the linear isomer (R3). Likewise, an analogous trend is discerned for the isomers of butyl butyrate, whereas the energy barriers of the β-branched isomers (R32) are 1.66 kcal/mol higher than that of the linear isomer (R39). This implies that the steric hindrance in the β-branched isomer may surpass the electronic effect, resulting in a less stable transition state.

In conclusion, the reactivity within this reaction class is predominantly independent of the overall carbon chain length. Nevertheless, it exhibits a high degree of sensitivity to the degree of the branching of alkyl groups. Branching at the α-position serves as a crucial determinant for augmented reactivity, which can be attributed to transition station stabilization. Conversely, branching at the β-position may marginally impede the reaction.

### 2.3. Reaction Enthalpies

**Figure 5:**
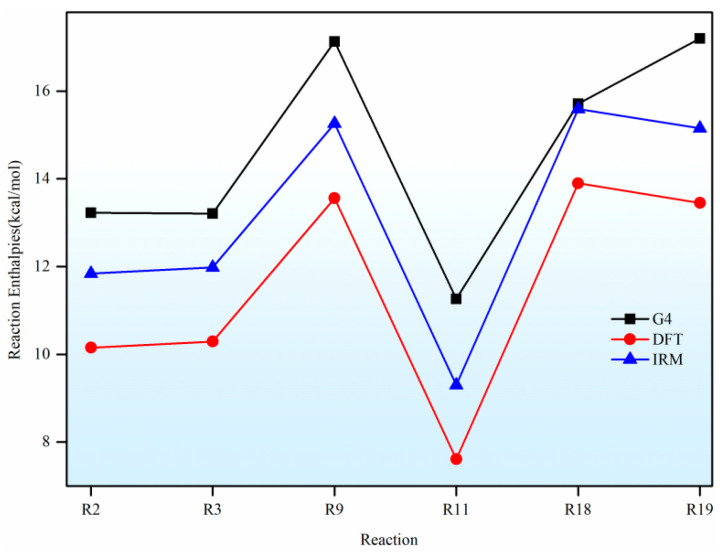
The reaction enthalpies calculated by the B3LYP, G4, and isodesmic reaction method (IRM).

In the present study, the reaction enthalpies for the unimolecular alkene elimination reaction class of fatty acid alkyl esters (FAAEs) were computed. To validate the correction scheme within the isodesmic reaction method, the reaction enthalpies obtained from the B3LYP, G4, and isodesmic reaction method (IRM) were compared, as depicted in Figure 5. As shown in Figure 5, it is evident that the discrepancies in reaction enthalpies calculated by the B3LYP method, relative to those determined by the G4 method, span from 1.81 to 3.75 kcal·mol^−1^. This indicates a notable difference between the two computational approaches. However, through the implementation of a correction scheme within the isodesmic reaction method, the discrepancies are significantly reduced. The adjusted deviations now range from 0.12 to 2.05 kcal·mol^−1^. This narrower range reflects a substantial enhancement in precision within the acceptable limits of chemical accuracy.

Based on the foregoing discussion, it can be deduced that the correction scheme within the isodesmic reaction method exhibits accuracy in calculating the energy barriers and reaction enthalpies of the unimolecular alkene elimination reaction category for fatty acid alkyl esters (FAAEs) at the low-level B3LYP method. Moreover, the energy barriers and reaction enthalpies of all reactions (calculated before and after correction via the isodesmic reaction method) are presented in detail in Table S2 of the Supplementary Materials.

### 2.4. Rate Coefficients and Rate Rules at High-Pressure-Limit

#### 2.4.1. Comparison of High-Pressure-Limit Rate Coefficients with Experiment Data

**Figure 6:**
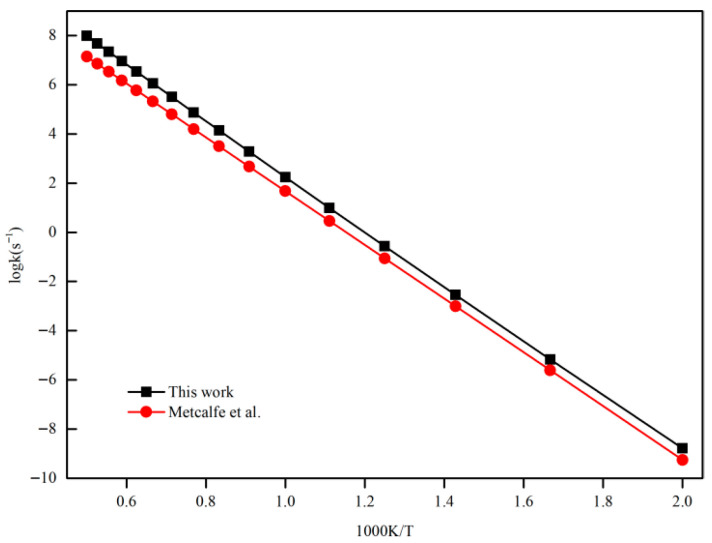
Comparison of the rate coefficients (s^−1^) at the high-pressure-limit to the literature data over the temperature range of 500~2000 K [9].

A direct comparison of the rate coefficients calculated in this work with experimental values is challenging due to the scarcity of experimental data that isolates the specific unimolecular alkene elimination pathway from other concurrent decomposition channels (e.g., C-C bond fission) in complex combustion environments. Only a few studies have been performed regarding the rate coefficients at the high-pressure-limit of the unimolecular elimination reactions for fatty acid alkyl esters. For instance, Metcalfe et al. [9] determined the rate coefficients for methyl propanoate. A comparison of these computed rate coefficients with existing research data is illustrated in Figure 6. Additionally, the rate coefficients calculated by us and those given by Metcalfe et al. [9] at temperatures of 500 K, 1000 K, 1500 K, and 2000 K are presented in Table 7.

It is evident from Figure 6 and Table 7 that for reaction R18, our calculated rate coefficients are close to those computed by Metcalfe et al. [9]; the ratio of our rate coefficients to those of Metcalfe et al. [9] ranges from 2.93 to 6.99. This level of discrepancy, within a single order of magnitude, is considered acceptable in computational kinetics for complex molecular systems.

#### 2.4.2. The Influence of Carbon Chain Length and the Effect of Branching for Substituent Groups on the Rate Coefficients

**Figure 7:**
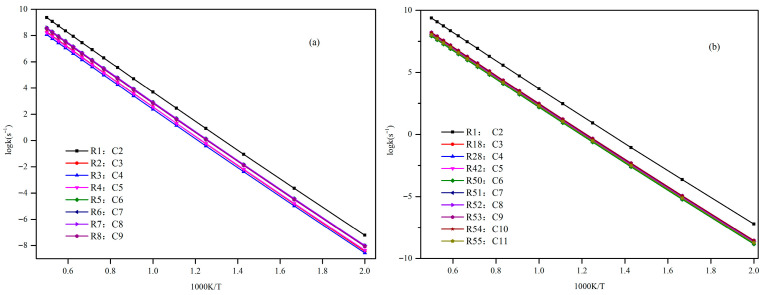
Comparison of the rate coefficients (s^−1^) at the high-pressure-limit between different carbon chain length over the temperature range of 500~2000 K. (**a**) acetic acid ester series; (**b**) ethyl ester series.

In this work, the impact of carbon chain length on the rate coefficients within the temperature range of 500~2000 K is presented in Figure 7. As depicted in Figure 7, for the acetates of linear alcohols (R1-R8) and the ethyl esters of linear carboxylic acids (R1, R18, R28, R42, R50–R55), in both cases, after an initial slight fluctuation from C2 to C4, the rate coefficients reach a stable state. For the acetate series (R3-R8), the values range from 2.39 × 10^2^ to 8.30 × 10^2^ s^−1^ at 1000 K, suggesting the length of the carbon chain has little effect on the rate coefficients. Similarly, for the ethyl ester series, the rate coefficients remain within a narrow range of approximately 1.53 × 10^2^~3.01 × 10^2^ s^−1^ at 1000 K. This notable consistency across a wide range of chain lengths (up to C11) provides conclusive evidence that the transition state is localized. The distal segments of long alkyl chains merely act as non-participating entities, exerting no significant electronic or steric influence that could alter the rate coefficients.

**Figure 8:**
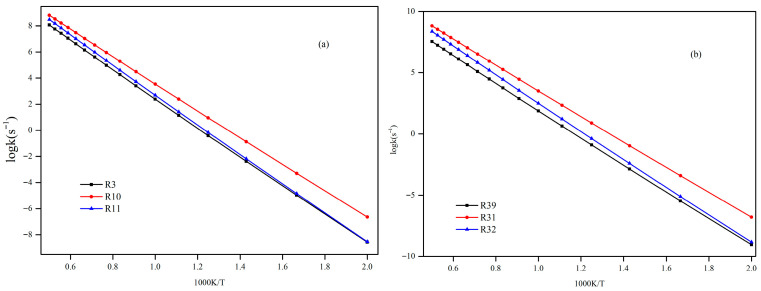
Comparison of the rate coefficients (s^−1^) at the high-pressure-limit between different branched isomers over the temperature range of 500~2000 K. (**a**) acetic acid butyl ester; (**b**) butyric acid butyl ester.

Furthermore, the influence of the degree of branching for alkyl chain on the rate coefficients was investigated through a comparison of linear, α-branched, and β-branched isomers within the temperature range of 500~2000 K, which are listed in Figure 8. The results indicate that the rate coefficients of the α-branched isomers (R10, R31) are higher than those of the linear isomers (R3, R39). This tendency is also in line with the reduction in the energy barrier discussed earlier. Nevertheless, a notably perceptive trend is discerned for the β-branched isomer, R11 and R32. Although it demonstrates a slightly higher energy barrier in comparison to its linear analog, R3 and R39, the calculated rate coefficients are comparable or even marginally larger throughout the investigated temperature range.

To quantify this phenomenon, the ratio of rate coefficients (k_branched_/k_linear_) was calculated, as shown in Table 8. The data in Table 8 clearly indicate that α-branching results in a substantial enhancement of the rate coefficients. The rate coefficients of the α-branched ester R10 are 48, 23, and 15 times higher than those of the linear ester R3 at 600 K, 800 K, and 1000 K, respectively. For other pairs, the rate coefficients enhancement is even more remarkable, with the k(R31)/k(R39) ratio exceeding a factor of 100 at 600 K. The significant acceleration induced by α-branching can be ascribed to the enhanced electron-donating inductive effect of the branched alkyl group, which effectively stabilizes the cyclic transition state. The results of the β-branching effect confirm that the calculated rate coefficients are comparable or even slightly larger across the investigated temperature range. This observation suggests that the steric hindrance in the β-branched isomer might outweigh the electronic effect, thereby giving rise to a less stable transition state. Additionally, the rate coefficient is also affected by temperature and the pre-exponential factor, which may be due to their combined effects.

#### 2.4.3. Rate Rules at the High-Pressure-Limit of Unimolecular Alkene Elimination for Fatty Acid Alkyl Esters

It is widely recognized that reaction mechanisms for large-size molecules are typically developed automatically by software based on the reaction rate rules. For each reaction class, only the smallest reaction system (namely the reaction with the smallest molecular size) in the same reaction class is employed to derive the rate rules. Nevertheless, in the study of Yao et al. [17], the results demonstrate that this method will introduce significant uncertainty into the rate rules. In the present work, the high-pressure-limit rate rules for reaction classes are derived by averaging the rate coefficients of a representative group of reactions with varying substituent group sizes and carbon chain lengths. The rate rules at the high-pressure-limit presented herein have been developed using a series of reactants containing C4~C13 fatty acid alkyl esters. Additionally, in software utilized for combustion modeling, including Chemkin-PRO, kinetic parameters are generally inputted in the form of a modified Arrhenius equation. Thus, the reaction kinetic parameters (A, n, E) for rate coefficients at the high-pressure-limit from 500 to 2000 K for all reactions are listed in Table S3 of the Supplementary Materials. To quantify the deviation between the rate coefficients predicted by the rate rule method and those from the quantum chemical method, an uncertainty factor *f* = *k_max_/k_min_* (where *k_max_ = k_IRM_*, *k_rul_*_e_ ; *k_min_ = k_IRM_, k_rul_*_e_) is defined as the ratio of the rate coefficients calculated via the isodesmic reaction method (IRM) to those predicted by the rate rules method for all reactions at 500 K and 1000 K. These *f* are also provided in Table S3 of the Supplementary Materials. The uncertainty factor *f* ranges from 1.21 to 59.5 at 500 K and from 1.07 to 12.9 at 1000 K. It is indicated that the uncertainty factor *f* of the rate rules decreases as the temperature increases. Consequently, the rate rules method provides a rapid approach for the mechanism generation program to determine the rate coefficients of large-molecular systems during the creation of the reaction mechanism for large molecules.

## 3. Methods

All B3LYP level electronic structure calculations were carried out with the BDF software 2025A [18,19,20,21,22], whereas high-level CCSD method and G4 method calculation were conducted via the Gaussian 16 software [23]. The B3LYP hybrid density functional, along with the 6-311G(d, p) basis set, was utilized to optimize the geometries of all species (reactants, transition states, and products) and as the low-level ab initio method within the isodesmic reaction correction scheme. The Gaussian-4 (G4) composite method serves as the high-level ab initio method within the isodesmic reaction correction scheme. It should be note that the G4 energies for transition states were obtained via single-point calculations performed on the fixed geometries optimized at the B3LYP/6-311G(d,p) level. For esters with long alkyl chains, structures were initialized in an extended conformation. Given the localized nature of the reaction, the energetics are expected to be insensitive to the conformation of the carbon chain. The transition states were located using the standard TS (transition state) optimization method. Intrinsic reaction coordinate (IRC) analyses [16,24] were conducted for all transition states to confirm that each correctly connects the reactants and products as the appropriate minima. Analytical harmonic frequency calculations were conducted at the B3LYP/6-311G(d,p) level to verify the presence of transition states with precisely one imaginary frequency, and other stationary geometries correspond to true local minimum with no imaginary frequencies. The harmonic frequencies were scaled by a factor of 0.97 for the calculation of thermodynamic properties [25]. All thermodynamic calculations were performed under the ideal gas approximation. The atomization method was used to calculate the standard enthalpies of formation Δ_f_H^θ^(298 K), and the experimental Δ_f_H^θ^(0 K) values for C (169.98 kcal·mol^−1^), H (51.63 kcal·mol^−1^), and O (58.99 kcal·mol^−1^) were utilized [26].

In the present work, the rate coefficients at the high-pressure-limit were calculated via the reaction class-transition state theory with the ChemRate program 1.5.8 [27]. The Eckart method [28] was utilized to account for the quantum mechanical tunneling effect. For traditional transition state theory [29], the rate coefficients can be expressed as follows:

(1) $$k\left(T\right)=\kappa (T)\sigma \frac{k_{B}T}{h}\frac{Q^{\neq }}{Q^{R}}exp\left(-\frac{{\Delta V}^{\neq }}{RT}\right)$$

where *k* is the tunneling coefficient; σ is the reaction symmetry number [30]; *k_B_* is Boltzmann constant; *T* is temperature; *h* is the Planck constant; *Q^≠^* and *Q^R^* are the total partition functions (per unit volume) of the transition state and the reactant, respectively, including translational, rotational, vibrational contributions; Δ*V^≠^* is the energy barrier, i.e., the difference in the electronic energies between the transition state and the reactant; and *R* is the ideal gas constant.

As usual, for small-size molecules (including C0~C4), a combination of high-level ab initio methods and traditional transition state theory is employed to obtain the rate coefficients. The reaction class-transition state theory (RC-TST), developed by Truong et al. [31,32,33], has been introduced into the dynamics calculations for large-size molecules. The reaction class-transition state theory, which builds on traditional transition state theory (TST), posits that all reactions within the same reaction class share the same reaction center, meaning there are certain similarities in the potential energy surface along the reaction coordinate. In RC-TST, a small-sized system within the reaction class is typically selected as the reference reaction, while other reactions in the class serve as target reactions. Then, the rate coefficients for reference reaction can be calculated from first principles using an accurate dynamical theory, with potential energy information computed from a sufficiently high-level of electronic structure theory, and the rate coefficients of target reactions, calculated using a relatively low-level ab initio method, can be corrected to yield results comparable to those from high-level ab initio calculations. Wang [34] and collaborators also provided a clear explanation of this method, which is called the isodesmic reaction method. For the isodesmic reaction method, the selection of an appropriate reference reaction is guided by three key criteria: (1) allowing for energy barrier and rate coefficient calculations at a high-level of theory; (2) ensuring that the reaction center contains the nature of the entire reaction process; and (3) the availability of reliable experimental data for validation. Based on these criteria, the reaction R1 from Table 1 was chosen as the reference for this study.

In the isodesmic reaction method, the reference reaction and the target reaction, in the process of forming their respective transition states, are defined as follows:

(2) $$A_{R}\to {TS}_{R}\;\;{\Delta V}_{R}$$

(3) $$A_{T}\to {TS}_{T}\;\;{\Delta V}_{T}$$

Herein, ${\Delta V}_{R}$ and ${\Delta V}_{T}$ are the energy barriers for the reference reaction (2) and the target reaction (3), respectively. According to the isodesmic reaction method, the difference between two reactions within a reaction class can also be regarded as an isodesmic reaction, such as reaction (4).

(4) $$A_{R}+{TS}_{T}\to A_{T}+{TS}_{r}\;\Delta \Delta V={\Delta V}_{R}-{\Delta V}_{T}$$

Here, ΔΔV represents the energy barriers as per the isodesmic reaction method. The values calculated using different ab initio methods exhibit little dependence on the theoretical level, primarily owing to the systematic cancelation of errors stemming from inadequate handling of electron correlation and incompleteness basis sets. The difference in energy barriers for the reference reaction between a low-level (B3LYP) method and a high-level (G4) method can then be expressed as Equation (5) for reference reaction, while Equation (6) (referred to as the isodesmic reaction correction scheme) enables the obtainment of a high-precision energy barrier from low-level ab initio calculations for target reactions. Similarly, accurate reaction enthalpies for target reactions can be derived using Equation (8).

(5) $${\Delta \Delta V}_{R}={\Delta V}_{R}(high)-{\Delta V}_{R}(low)$$

(6) $${\Delta V}_{T}\left(high\right)={\Delta \Delta V}_{R}+{\Delta V}_{T}(low)$$

(7) $${\Delta \Delta H}_{R}={\Delta H}_{R}(high)-{\Delta H}_{R}(low)$$

(8) $${\Delta H}_{T}\left(high\right)={\Delta \Delta H}_{R}+{\Delta H}_{T}(low)$$

In accordance with the isodesmic reaction correction scheme, the accurate rate coefficients *k*′ for target reactions can be obtained by adjusting the approximate rate coefficients *k* at a low ab initio level for target reactions using the expression below:

(9) $$k^{\prime }=kexp\left(-\frac{{\Delta \Delta V}^{\prime }}{RT}\right)$$

where *k* is the rate coefficients for target reaction calculated at the low-level (B3LYP) ab initio method, and *k*′ is the corrected rate coefficients for the target reaction within the isodesmic reaction method. ΔΔV is the correction scheme for energy barriers from the reference reaction in Equation (5).

## 4. Conclusions

In this paper, a correction scheme within the isodesmic reaction method is utilized to correct the energy barriers, reaction enthalpies, and rate coefficients at the B3LYP level corresponding to the unimolecular alkene elimination reaction class of fatty acid alkyl esters (FAAEs). When comparing the corrected energy barriers and reaction enthalpies with the B3LYP results for a set of target reactions in the class (at the G4 level), it is found that the corrected outcomes are close to the values obtained using the G4 method. Furthermore, the rate coefficients obtained in this paper are comparable to experiment data, demonstrating that the energy barriers, reaction enthalpies, and rate coefficients are acceptable, and that the proposed isodesmic reaction correction scheme represents a viable approach for the accurate calculation of kinetic parameters for large-size molecules. Additionally, the rate rules at the high-pressure-limit are derived by averaging the rate coefficients of a representative set of reactions with different substituent sizes and carbon chain length. The findings show that the uncertainty factor *f* ranges from 1.21 to 59.5 at 500 K and from 1.07 to 12.9 at 1000 K, indicating that the uncertainty factor *f* of the rate rules decreases as the temperature increases. Consequently, the rate rules method provides a rapid approach for the mechanism generation program to determine the rate coefficients of large-molecular systems during the creation of the reaction mechanism for large molecules.

Furthermore, the impacts of the carbon chain length and the branching degree of alkyl groups on the energy barriers and rate coefficients are systematically examined. The results show that as the carbon chain length increases, the change in energy barrier is small. It is attributed to the distal segments of long alkyl chains merely act as non-participating entities, exerting no significant electronic or steric influence that could alter the energy barriers and rate coefficients. Nevertheless, it exhibits a high degree of sensitivity to the degree of the branching on energy barriers, wherein the α-branching can lower the energy barrier, while β-branching leads to an increase in the energy barrier. Meanwhile, the degree of branching in the alkyl chain exerts a profound influence on the rate coefficients. The α-branching consistently provides a significant kinetic advantage by lowering the activation barrier, resulting in a rate acceleration across 500~2000 K. In contrast, the effect of β-branching is markedly weaker. The significant acceleration induced by α-branching can be ascribed to the enhanced electron-donating inductive effect of the branched alkyl group, which effectively stabilizes the cyclic transition state. The results of the β-branching effect confirm that the calculated rate coefficients are comparable or even slightly larger across the investigated temperature range. This observation suggests that the steric hindrance in the β-branched isomer might outweigh the electronic effect, thereby giving rise to a less stable transition state. Additionally, the rate coefficient is also affected by temperature and the pre-exponential factor, which may be due to their combined effects.

In this study, a unified high-level theoretical method has been applied for the first time to a wide range of large-size fatty acid alkyl ester (FAAEs) molecules to obtain accurate kinetic parameters for this reaction class. This allows combustion modelers to incorporate these previously unaccounted-for reaction pathways into reaction mechanisms with a quantifiable level of certainty, directly addressing a well-acknowledged deficiency in biodiesel combustion models. The data presented here will promote more accurate simulations of biodiesel decomposition, thus contributing to a more profound fundamental understanding of the process and its results. The comprehensive kinetic dataset provided in this work establishes a fundamental basis for understanding structural effects in complex reactions in the reaction mechanism of biodiesel fuels, with extensive implications for predicting molecular reactivity and guiding the synthesis of target molecules with tailored kinetic characteristics.

## Figures and Tables

**Table 1:** Reaction lists.

Reaction Class	Reaction Equation
R1	CH_3_COOC_2_H_5_→CH_3_COOH + CH_2_ = CH_2_
R2	CH_3_COOC_3_H_7_→CH_3_COOH + CH_2_ = CHCH_3_
R3	CH_3_COOC_4_H_9_→CH_3_COOH + CH_2_ = CHC_2_H_5_
R4	CH_3_COOC_5_H_11_→CH_3_COOH + CH_2_ = CHC_3_H_7_
R5	CH_3_COOC_6_H_13_→CH_3_COOH + CH_2_ = CHC_4_H_9_
R6	CH_3_COOC_7_H_15_→CH_3_COOH + CH_2_ = CHC_5_H_11_
R7	CH_3_COOC_8_H_17_→CH_3_COOH + CH_2_ = CHC_6_H_13_
R8	CH_3_COOC_9_H_19_→CH_3_COOH + CH_2_ = CHC_7_H_15_
R9	CH_3_COOCH(CH_3_)CH_3_→CH_3_COOH + CH_3_CH = CH_2_
R10	CH_3_COOCH(CH_3_)C_2_H_5_→CH_3_COOH + CH_3_CH = CHCH_3_
R11	CH_3_COOCH_2_CH(CH_3_)CH_3_→CH_3_COOH + CH_2_ = C(CH_3_)CH_3_
R12	CH_3_COOCH_2_CH(C_2_H_5_)CH_3_→CH_3_COOH + CH_2_ = C(C_2_H_5_)CH_3_
R13	CH_3_COOCH(CH_3_)CH(CH_3_)CH_3_→CH_3_COOH + CH_3_CH = C(CH_3_)CH_3_
R14	CH_3_COOCH(CH_3_)C_3_H_7_→CH_3_COOH + CH_2_ = CHC_3_H_7_
R15	CH_3_COOCH_2_CH(CH_3_)C_2_H_5_→CH_3_COOH + CH_2_ = C(CH_3_)C_2_H_5_
R16	CH_3_COOCH_2_CH(C_2_H_5_)C_2_H_5_→CH_3_COOH + CH_2_ = C(C_2_H_5_)C_2_H_5_
R17	CH_3_COOCH_2_CH_2_CH(CH_3_)CH_3_→CH_3_COOH + CH_2_ = CHCH(CH_3_)CH_3_
R18	C_2_H_5_COOC_2_H_5_→C_2_H_5_COOH + CH_2_ = CH_2_
R19	C_2_H_5_COOCH(CH_3_)CH_3_→C_2_H_5_COOH + CH_2_ = CHCH_3_
R20	C_2_H_5_COOCH(CH_3_)C_2_H_5_→C_2_H_5_COOH + CH_3_CH = CHCH_3_
R21	C_2_H_5_COOCH_2_CH(CH_3_)CH_3_→C_2_H_5_COOH + CH_2_ = C(CH_3_)CH_3_
R22	C_2_H_5_COOCH_2_CH(C_2_H_5_)CH_3_→C_2_H_5_COOH + CH_2_ = C(C_2_H_5_)CH_3_
R23	C_2_H_5_COOCH(CH_3_)CH(CH_3_)CH_3_→C_2_H_5_COOH + CH_3_CH = C(CH_3_)CH_3_
R24	C_2_H_5_COOCH(CH_3_)C_3_H_7_→C_2_H_5_COOH + CH_2_ = CHC_3_H_7_
R25	C_2_H_5_COOCH_2_CH(CH_3_)C_2_H_5_→C_2_H_5_COOH + CH_2_ = C(CH_3_)C_2_H_5_
R26	C_2_H_5_COOCH_2_CH(C_2_H_5_)C_2_H_5_→C_2_H_5_COOH + CH_2_ = C(C_2_H_5_)C_2_H_5_
R27	C_2_H_5_COOCH_2_CH_2_CH(CH_3_)CH_3_→C_2_H_5_COOH + CH_2_ = CHCH(CH_3_)CH_3_
R28	C_3_H_7_COOC_2_H_5_→C_3_H_7_COOH + CH_2_ = CH_2_
R29	C_3_H_7_COOC_3_H_7_→C_3_H_7_COOH + CH_2_ = CHCH_3_
R30	C_3_H_7_COOCH(CH_3_)CH_3_→C_3_H_7_COOH + CH_3_CH = CH_2_
R31	C_3_H_7_COOCH(CH_3_)C_2_H_5_→C_3_H_7_COOH + CH_3_CH = CHCH_3_
R32	C_3_H_7_COOCH_2_CH(CH_3_)CH_3_→C_3_H_7_COOH + CH_2_ = C(CH_3_)CH_3_
R33	C_3_H_7_COOCH_2_CH(C_2_H_5_)CH_3_→C_3_H_7_COOH + CH_2_ = C(C_2_H_5_)CH_3_
R34	C_3_H_7_COOCH(CH_3_)CH(CH_3_)CH_3_→C_3_H_7_COOH + CH_3_CH = C(CH_3_)CH_3_
R35	C_3_H_7_COOCH(CH_3_)C_3_H_7_→C_3_H_7_COOH + CH_2_ = CHC_3_H_7_
R36	C_3_H_7_COOCH_2_CH(CH_3_)C_2_H_5_→C_3_H_7_COOH + CH_2_ = C(CH_3_)C_2_H_5_
R37	C_3_H_7_COOCH_2_CH(C_2_H_5_)C_2_H_5_→C_3_H_7_COOH + CH_2_ = C(C_2_H_5_)C_2_H_5_
R38	C_3_H_7_COOCH_2_CH_2_CH(CH_3_)CH_3_→C_3_H_7_COOH + CH_2_ = CHCH(CH_3_)CH_3_
R39	C_3_H_7_COOC_4_H_9_→C_3_H_7_COOH + CH_2_ = CHC_2_H_5_
R40	C_3_H_7_COOC_5_H_11_→C_3_H_7_COOH + CH_2_ = CHC_3_H_7_
R41	C_3_H_7_COOC_6_H_13_→C_3_H_7_COOH + CH_2_ = CHC_4_H_9_
R42	C_4_H_9_COOC_2_H_5_→C_4_H_9_COOH + CH_2_ = CH_2_
R43	C_4_H_9_COOC_3_H_7_→C_4_H_9_COOH + CH_2_ = CHCH_3_
R44	C_4_H_9_COOC_4_H_9_→C_4_H_9_COOH + CH_2_ = CHC_2_H_5_
R45	C_4_H_9_COOC_5_H_11_→C_4_H_9_COOH + CH_2_ = CHC_3_H_7_
R46	C_5_H_11_COOC_3_H_7_→C_5_H_11_COOH + CH_2_ = CHCH_3_
R47	C_5_H_11_COOC_4_H_9_→C_5_H_11_COOH + CH_2_ = CHC_2_H_5_
R48	C_5_H_11_COOC_5_H_11_→C_5_H_11_COOH + CH_2_ = CHC_3_H_7_
R49	C_5_H_11_COOC_6_H_13_→C_5_H_11_COOH + CH_2_ = CHC_4_H_9_
R50	C_5_H_11_COOC_2_H_5_→C_5_H_11_COOH + CH_2_ = CH_2_
R51	C_6_H_13_COOC_2_H_5_→C_6_H_13_COOH + CH_2_ = CH_2_
R52	C_7_H_15_COOC_2_H_5_→C_7_H_15_COOH + CH_2_ = CH_2_
R53	C_8_H_17_COOC_2_H_5_→C_8_H_17_COOH + CH_2_ = CH_2_
R54	C_9_H_19_COOC_2_H_5_→C_9_H_19_COOH + CH_2_ = CH_2_
R55	C_10_H_21_COOC_2_H_5_→C_10_H_21_COOH + CH_2_ = CH_2_

**Table 2:** Deviation of the geometrical parameters for reaction center in transition states.

	d_1_/Å	A_1_/(°)	d_2_/Å	A_2_/(°)	d_3_/Å	A_3_/(°)	d_4_/Å	A_4_/(°)	d_5_/Å	A_5_/(°)	d_6_/Å	A_6_/(°)
^a^ Avg	1.26	123.77	2.04	115.69	1.41	110.43	1.30	94.42	1.34	169.89	1.27	103.44
^b^ Mad	0.00	0.90	0.15	1.60	0.02	4.30	0.08	5.50	0.12	6.40	0.00	2.50

^a^ Average values of geometrical parameters corresponding to transition states. ^b^ Maximum absolute deviation of geometrical parameters across different reactions.

**Table 3:** Comparison of the energy barriers (in kcal·mol^−1^) between the B3LYP and B3LYP-D3 level of theory.

Reaction	B3LYP	B3LYP-D3	Δ*V*
R9	44.84	44.35	0.49
R22	48.38	47.50	0.88
R38	48.07	46.62	1.45
R49	48.98	47.04	1.94
R55	48.79	47.96	0.83
^a^ MAD			1.12

^a^ MAD represents the mean absolute deviation of the differences in energy barriers between B3LYP and B3LYP-D3.

**Table 4:** Reaction enthalpy and energy barrier for reference reaction R1 (kcal·mol^−1^).

Reaction	Δ*V*		^a^ ΔΔ*V*	Δ*H*		^b^ ΔΔ*H*
	G4	B3LYP		G4	B3LYP	
R1	54.58	48.27	6.31	15.70	14.01	1.69

^a^ Difference in energy barriers between the G4 method and the B3LYP method. ^b^ Difference in reaction enthalpies between the G4 method and the B3LYP method.

**Table 5:** Comparison of the energy barriers (${\Delta V}^{\neq }$) before and after correction with the G4 method (kcal·mol^−1^).

Reaction	Δ*V*		
	B3LYP	G4	^a^ IRM
R2	48.64 (6.50) *	55.14	54.95 (0.19) ^#^
R3	48.39 (5.99)	54.38	54.70 (−0.32)
R9	44.84 (7.12)	51.96	51.15 (0.81)
R11	49.40 (6.20)	55.60	55.71 (−0.11)
R18	48.85 (6.16)	55.01	55.16 (−0.15)
R19	45.35 (7.09)	52.41	51.66 (0.75)

^a^ Energy barriers derived from the B3LYP method after correction via the isodesmic reaction method (IRM). * The values in parentheses are the deviations of the energy barriers between the G4 method and the B3LYP method. ^#^ The values in parentheses are the deviations of the energy barriers between the G4 method and the isodesmic reaction method (IRM).

**Table 6:** Comparison of the energy barriers for straight-chain, α-branched, and β-branched isomers.

Alkyl Chain	Reaction	Substituent Groups Type	Energy Barrier (kcal·mol^−1^)
Propyl	R2	linear	54.95
	R9	α-branched	51.15 (3.80) ^a^
Butyl	R3	linear	54.70
	R10	α-branched	50.79 (3.91)
	R11	β-branched	55.71 (1.01)
Butyl	R39	linear	54.55
	R31	α-branched	51.25 (3.30)
	R32	β-branched	56.21 (1.66)

^a^ The values in the brackets represent the difference between the energy barriers of the linear and the branched chain isomers.

**Table 7:** Comparison of the rate coefficients (s^−1^) at high-pressure-limit with the literature data.

C_2_H_5_COOC_2_H_5_→C_2_H_5_COOH + CH_2_ = CH_2_				
T/K	500	1000	1500	2000
This work	1.63 × 10^−9^	1.75 × 10^2^	1.13 × 10^6^	9.61 × 10^7^
Metcalfe et al. [9]	5.57 × 10^−10^ (2.93) ^a^	4.72 × 10 (3.72)	2.07 × 10^5^ (5.43)	1.37 × 10^7^ (6.99)

^a^ Values in parentheses are the ratio of the rate coefficients of this work to the results of Metcalfe et al. [9].

**Table 8:** Comparison of the rate coefficients (s^−1^) between distinct branched isomers and linear isomers.

Isomer Type	Reaction	*k*		
		600 K	800 K	1000 K
Linear (Propyl)	R2	1.46 × 10^−5^	5.73 × 10^−1^	3.62 × 10^2^
α-Branched	R9	1.37 × 10^−4^	2.58	1.03 × 10^3^
Ratio (R9/R2)		9.37	4.50	2.86
Linear (Propyl)	R3	1.05 × 10^−5^	3.90 × 10^−1^	2.39 × 10^2^
α-Branched	R10	5.05 × 10^−4^	9.07	3.51 × 10^3^
β-Branched	R11	1.41 × 10^−5^	6.79 × 10^−1^	4.82 × 10^2^
* Ratio (R10/R3)		4.82 × 10^1^	2.32 × 10	1.47 × 10
* Ratio (R11/R3)		1.34	1.74	2.02
Linear (Propyl)	R39	3.42 × 10^−6^	1.24 × 10^−1^	7.46 × 10
α-Branched	R31	3.90 × 10^−4^	7.72	3.17 × 10^3^
β-Branched	R32	7.66 × 10^−6^	4.15 × 10^−1^	3.16 × 10^2^
Ratio (R31/R39)		1.14 × 10^2^	6.23 × 10	4.25 × 10
Ratio (R32/R39)		2.24	3.35	4.23

* The ratio of the rate coefficients between different branched isomers with linear isomers.

## Data Availability

Data are contained within the article and Supplementary Materials.

## Supplementary Materials

The following supporting information can be downloaded at: https://www.mdpi.com/article/10.3390/molecules30204054/s1. The geometric parameters of the reaction center optimized at B3LYP/6-311G(d,p) level are listed in Table S1. Optimized geometrical parameters of the reaction-center for transition states; Table S2. Energy barriers and reaction enthalpies before and after correction by the isodesmic reaction method for all reactions; Table S3. High-pressure-limit rate rules, ratio of the rate coefficients and kinetic parameters (A, n, E) for unimolecular elimination reaction class of fatty acid alkyl esters; Table S4. Cartesian coordinates for all transition states.
