# Supplementary material for: Kinetic Parameters at High-Pressure-Limit for Unimolecular Alkene Elimination Reaction Class of Fatty Acid Alkyl Esters (FAAEs)
Source: Molecules. 2025 Oct 11;30(20):4054. doi: 10.3390/molecules30204054 (PMC12565754; doi:10.3390/molecules30204054)
Supplement: Supplementary file 1 [file molecules-30-04054-s001.zip › molecules-3867244-supplementary.pdf]

Supplementary Material

Kinetic Parameters at High-Pressure-Limit for Unimolecular Alkene Elimination Reaction Class of Fatty Acid Alkyl Esters (FAAE)

Xiao-Hui Sun[1,*], Zhen-Yu Pei[1], Ze-Rong Li[2,*], Yuan-Yuan Tian[1]

1 School of Energy Engineering, Shanxi College of Technology, Shuozhou 036000, China; peizhenyu@sxct.edu.cn (Z.P.); tianyuanyuan@sxct.edu.cn (Y.T.)

2 College of Chemistry, Sichuan University, Chengdu 610064, China

* Correspondence: xiaohuisun_sxct@163.com (X.S.); lizerong@scu.edu.cn (Z.L.)

Content

Table S1. Optimized geometrical parameters of the reaction-center for transition states

Table S2. Energy barriers and reaction enthalpies before and after correction by the isodesmic reaction method for all reactions

Table S3. High-pressure-limit rate rules, ratio of the rate coefficients and kinetic parameters (A, n, E) for unimolecular elimination reaction class of fatty acid alkyl esters.

Table S4. Cartesian coordinates for all transition states

Table S1. Optimized geometrical parameters of the reaction center for transition states.

TS	d_1/Å	A_1/(^{0})	d_2/Å	A_2/(^{0})	d_3/Å	A_3/(^{0})	d_4/Å	A_4/(^{0})	d_5/Å	A_5/(^{0})	d_6/Å	A_6/(^{0})
TS1	1.26	124.30	1.99	116.50	1.40	111.10	1.33	95.90	1.30	167.60	1.27	104.60
TS2	1.26	124.10	2.02	115.80	1.40	111.10	1.30	94.20	1.33	169.10	1.27	103.50
TS3	1.26	124.10	2.02	115.90	1.40	111.10	1.30	94.40	1.33	169.10	1.27	103.60
TS4	1.26	124.10	2.02	115.80	1.40	111.10	1.30	94.40	1.33	169.00	1.27	103.60
TS5	1.26	124.10	2.02	115.90	1.40	111.10	1.30	94.40	1.33	169.10	1.27	103.60
TS6	1.26	124.10	2.02	115.80	1.40	111.10	1.30	94.40	1.33	169.00	1.27	103.60
TS7	1.26	124.10	2.02	115.80	1.40	111.10	1.30	94.40	1.33	169.10	1.27	103.60
TS8	1.26	124.10	2.02	115.90	1.40	111.10	1.30	94.40	1.33	169.10	1.27	103.60
TS9	1.26	124.00	2.09	116.10	1.41	107.50	1.29	97.80	1.34	168.70	1.27	104.00
TS10	1.26	123.90	2.12	115.40	1.41	107.50	1.27	95.80	1.38	169.50	1.27	103.00
TS11	1.26	124.10	2.03	115.80	1.41	111.70	1.28	92.50	1.36	172.90	1.27	102.70
TS12	1.26	124.10	2.03	115.70	1.41	111.70	1.28	92.60	1.36	172.70	1.27	102.80
TS13	1.26	123.90	2.13	115.50	1.42	108.30	1.25	94.20	1.41	173.80	1.27	102.20
TS14	1.26	123.70	2.12	115.10	1.41	107.40	1.27	95.70	1.38	169.00	1.27	102.80
TS15	1.26	123.80	2.04	115.20	1.41	111.70	1.28	92.50	1.37	172.60	1.27	102.50
TS16	1.26	123.80	2.04	115.20	1.41	111.60	1.28	92.60	1.37	172.40	1.27	102.50
TS17	1.26	123.80	2.03	115.40	1.40	110.90	1.30	94.20	1.33	168.70	1.27	103.40
TS18	1.26	123.90	2.00	116.50	1.40	111.00	1.33	95.80	1.29	167.80	1.27	104.60
TS19	1.26	123.70	2.10	116.00	1.41	107.40	1.30	97.70	1.34	168.90	1.27	104.10

TS20	1.26	123.60	2.12	115.30	1.41	107.50	1.27	95.60	1.37	169.60	1.27	103.00
TS21	1.26	123.80	2.04	115.60	1.41	111.70	1.28	92.40	1.36	173.10	1.27	102.70
TS22	1.26	123.70	2.04	115.50	1.41	111.60	1.28	92.50	1.36	172.80	1.27	102.70
TS23	1.26	123.70	2.14	115.20	1.42	108.20	1.26	94.10	1.40	174.00	1.27	102.20
TS24	1.26	123.50	2.13	115.00	1.41	107.40	1.27	95.60	1.38	169.20	1.27	102.90
TS25	1.26	123.50	2.05	115.10	1.41	111.60	1.29	92.30	1.36	172.80	1.27	102.50
TS26	1.26	123.50	2.05	115.00	1.41	111.60	1.28	92.40	1.36	172.60	1.27	102.50
TS27	1.26	123.50	2.03	115.30	1.40	110.90	1.31	94.10	1.33	168.70	1.27	103.40
TS28	1.26	123.90	1.99	116.60	1.40	111.10	1.33	95.80	1.29	167.70	1.27	104.70
TS29	1.26	123.90	2.02	116.10	1.40	111.20	1.31	94.00	1.33	169.90	1.27	103.90
TS30	1.26	123.80	2.10	116.40	1.41	107.50	1.29	97.70	1.34	169.10	1.27	104.30
TS31	1.26	123.50	2.12	115.40	1.41	107.50	1.27	95.60	1.37	169.60	1.27	103.10
TS32	1.26	123.70	2.04	115.70	1.41	111.70	1.28	92.40	1.36	173.10	1.27	102.80
TS33	1.26	123.70	2.04	115.60	1.41	111.70	1.28	92.50	1.36	172.80	1.27	102.80
TS34	1.26	123.60	2.14	115.30	1.42	108.20	1.25	94.10	1.41	173.90	1.27	102.30
TS35	1.26	123.40	2.13	115.10	1.41	107.40	1.27	95.60	1.38	169.20	1.27	103.00
TS36	1.26	123.50	2.05	115.20	1.41	111.70	1.29	92.30	1.36	172.70	1.27	102.60
TS37	1.26	123.50	2.05	115.10	1.41	111.60	1.28	92.50	1.36	172.60	1.27	102.60
TS38	1.26	123.50	2.03	115.40	1.40	110.90	1.31	94.10	1.33	168.70	1.27	103.60
TS39	1.26	123.50	2.03	115.40	1.40	111.00	1.31	94.10	1.33	168.70	1.27	103.60
TS40	1.26	123.50	2.03	115.30	1.40	111.00	1.31	94.00	1.33	168.60	1.27	103.50

TS41	1.26	123.50	2.03	115.30	1.40	110.90	1.31	94.00	1.33	168.60	1.27	103.50
TS42	1.26	123.90	1.99	116.60	1.40	111.10	1.33	95.80	1.29	167.70	1.27	104.70
TS43	1.26	123.70	2.02	115.80	1.40	111.10	1.31	94.00	1.33	169.20	1.27	103.60
TS44	1.26	123.50	2.03	115.40	1.40	111.00	1.31	94.10	1.33	168.70	1.27	103.60
TS45	1.26	123.70	2.03	115.60	1.40	111.00	1.31	94.00	1.33	169.00	1.27	103.70
TS46	1.26	123.70	2.02	115.70	1.40	111.00	1.31	94.00	1.33	169.10	1.27	103.60
TS47	1.26	123.50	2.03	115.30	1.40	110.90	1.31	94.00	1.33	168.60	1.27	103.50
TS48	1.26	123.50	2.03	115.40	1.40	111.00	1.31	94.00	1.33	168.60	1.27	103.60
TS49	1.26	123.50	2.03	115.40	1.40	111.00	1.31	94.00	1.33	168.70	1.27	103.60
TS50	1.26	123.90	1.99	116.60	1.40	111.10	1.33	95.80	1.29	167.70	1.27	104.70
TS51	1.26	123.90	1.99	116.60	1.40	111.10	1.33	95.80	1.29	167.70	1.27	104.70
TS52	1.26	123.90	1.99	116.60	1.40	111.10	1.33	95.80	1.29	167.70	1.27	104.70
TS53	1.26	123.90	1.99	116.60	1.40	111.10	1.33	95.80	1.29	167.70	1.27	104.70
TS54	1.26	123.90	1.99	116.60	1.40	111.10	1.33	95.80	1.29	167.70	1.27	104.70
TS55	1.26	124.30	1.99	116.50	1.40	111.10	1.33	95.90	1.30	167.60	1.27	104.60

Table S2. Energy barriers and reaction ehthalpies for all reactions (kcal/mol).

Reaction	${}^{a}\Delta V^{\neq}$	${}^{b}\Delta V^{\neq\prime}$	${}^{c}\Delta H^{\neq}$	${}^{d}\Delta H^{\neq\prime}$
R1	48.27	54.58	14.01	15.70
R2	48.64	54.95	10.15	11.84
R3	48.39	54.70	10.29	11.98
R4	48.27	54.58	10.22	11.92
R5	48.26	54.57	10.19	11.88
R6	48.26	54.57	10.19	11.89
R7	48.26	54.57	10.19	11.88
R8	48.28	54.59	10.19	11.88
R9	44.84	51.15	13.56	15.26
R10	44.48	50.79	9.61	11.30
R11	49.40	55.71	7.61	7.61
R12	47.92	54.23	6.59	8.28
R13	45.23	51.54	7.86	9.55
R14	44.25	50.56	12.79	14.48
R15	49.14	55.45	7.64	9.33
R16	47.34	53.65	7.43	9.13
R17	47.56	53.87	9.46	11.15
R18	48.85	55.16	13.90	15.59
R19	45.35	51.66	13.45	15.15
R20	44.96	51.27	9.48	11.18
R21	49.91	56.22	7.50	9.19
R22	48.38	54.69	6.45	8.14
R23	45.68	51.99	7.70	9.39
R24	44.74	51.05	12.68	14.38
R25	49.63	55.94	7.52	9.21
R26	47.83	54.14	7.28	8.97
R27	48.10	54.41	9.34	11.03
R28	48.81	55.12	13.91	15.60
R29	49.20	55.51	10.04	11.73

R30	45.39	51.70	13.44	15.14
R31	44.94	51.25	9.45	11.14
R32	49.90	56.21	7.51	9.20
R33	48.36	54.67	6.48	8.18
R34	45.63	51.94	7.68	9.37
R35	42.33	48.64	10.14	11.84
R36	48.76	55.07	6.57	8.27
R37	47.30	53.61	6.69	8.38
R38	48.07	54.38	9.37	11.06
R39	48.24	54.55	9.35	11.04
R40	48.20	54.51	9.29	10.99
R41	48.19	54.50	9.28	10.98
R42	48.79	55.10	13.89	15.58
R43	49.14	55.45	10.02	11.72
R44	49.01	55.32	10.16	11.86
R45	49.14	55.45	10.10	11.79
R46	49.13	55.44	10.02	11.71
R47	49.02	55.33	10.17	11.86
R48	48.98	55.29	10.10	11.80
R49	48.99	55.30	10.08	11.78
R50	48.80	55.11	13.89	15.58
R51	48.79	55.10	13.89	15.58
R52	48.79	55.10	13.87	15.57
R53	48.79	55.10	13.88	15.58
R54	48.80	55.11	13.87	15.56
R55	48.79	55.10	13.88	15.58

[a]The energy barriers before correction. [b]The energy barriers after correction.[c]The reaction enthalpies before correction. [d]The reaction enthalpies after correction.

Table S3. Ratio of the rate coefficients and kinetic parameters (A, n, E) for unimolecular alkene elimination reaction class of fatty acid alkyl esters.

Reaction	A/s^{-1}	n	E/(kcal/mol)	500K		1000K	
				k_{IRM}	f	k_{IRM}	f
R1	3.77E+09	1.53	47.86	6.23E-08	1.37	4.95E+03	5.14
R2	5.72E+08	1.46	48.41	3.61E-09	1.26E+01	3.62E+02	2.66
R3	3.26E+08	1.46	48.17	2.69E-09	1.69E+01	2.39E+02	4.03
R4	4.78E+08	1.46	48.04	4.28E-09	1.06E+01	3.56E+02	2.70
R5	1.13E+09	1.46	48.06	9.86E-09	4.62	8.27E+02	1.16
R6	8.92E+08	1.47	48.01	9.24E-09	4.93	7.64E+02	1.26
R7	1.01E+09	1.47	48.02	1.00E-08	4.55	8.30E+02	1.16
R8	8.04E+08	1.48	48.01	8.40E-09	5.42	6.97E+02	1.38
R9	1.25E+09	1.26	45.20	5.89E-08	1.29	1.03E+03	1.07
R10	6.97E+09	1.19	45.09	2.22E-07	4.88	3.51E+03	3.64
R11	2.85E+09	1.35	49.56	2.89E-09	1.58E+01	4.82E+02	2.00
R12	3.33E+09	1.36	48.07	1.56E-08	2.92	1.23E+03	1.28
R13	3.38E+10	1.11	46.09	2.52E-07	5.53	6.29E+03	6.53
R14	6.09E+09	1.17	44.89	2.23E-07	4.90	3.16E+03	3.28
R15	9.44E+08	1.35	49.30	1.24E-09	3.67E+01	1.82E+02	5.30
R16	6.16E+08	1.33	47.59	4.00E-09	1.14E+01	2.44E+02	3.95
R17	3.09E+08	1.46	47.30	6.07E-09	7.50	3.48E+02	2.77
R18	1.82E+08	1.53	48.47	1.63E-09	2.79E+01	1.75E+02	5.49
R19	1.93E+09	1.27	45.71	5.51E-08	1.21	1.26E+03	1.31
R20	4.28E+09	1.19	45.56	8.77E-08	1.93	1.76E+03	1.83
R21	2.35E+09	1.35	50.10	1.34E-09	3.40E+01	2.93E+02	3.29
R22	1.76E+09	1.35	48.61	4.42E-09	1.03E+01	4.56E+02	2.11
R23	2.74E+10	1.11	46.54	1.29E-07	2.84	4.03E+03	4.19
R24	4.50E+09	1.17	45.43	9.09E-08	2.00	1.69E+03	1.75
R25	1.30E+09	1.35	49.84	9.88E-10	4.60E+01	1.90E+02	5.08
R26	1.13E+08	1.55	47.82	2.17E-09	2.10E+01	1.72E+02	5.60
R27	2.93E+08	1.45	47.91	2.82E-09	1.62E+01	2.18E+02	4.41
R28	1.64E+08	1.52	48.46	1.48E-09	3.07E+01	1.59E+02	6.07
R29	4.94E+08	1.45	49.03	1.61E-09	2.82E+01	2.20E+02	4.73

R30	1.26E+09	1.26	45.73	3.38E-08	1.35	7.73E+02	1.25
R31	7.40E+09	1.19	45.53	1.59E-07	3.50	3.17E+03	3.29
R32	2.56E+09	1.35	50.11	1.44E-09	3.17E+01	3.16E+02	3.05
R33	7.07E+08	1.33	48.65	1.57E-09	2.90E+01	1.63E+02	5.90
R34	2.76E+10	1.11	46.56	1.23E-07	2.71	3.88E+03	4.03
R35	3.56E+09	1.16	42.92	8.67E-07	1.90E+01	4.53E+03	4.70
R36	8.11E+08	1.34	48.92	1.49E-09	3.06E+01	1.79E+02	5.37
R37	1.19E+09	1.34	47.56	8.20E-09	5.55	4.96E+02	1.94
R38	1.67E+08	1.43	47.94	1.45E-09	3.14E+01	1.13E+02	8.56
R39	1.08E+08	1.45	48.06	8.94E-10	5.09E+01	7.46E+01	1.29E+01
R40	3.30E+08	1.45	48.00	2.97E-09	1.53E+01	2.42E+02	3.98
R41	5.00E+08	1.45	48.02	4.41E-09	1.03E+01	3.60E+02	2.67
R42	1.90E+08	1.53	48.44	1.76E-09	2.59E+01	1.86E+02	5.17
R43	4.08E+08	1.46	48.95	1.48E-09	3.08E+01	1.94E+02	4.95
R44	1.92E+08	1.46	48.85	7.65E-10	5.95E+01	9.57E+01	1.01E+01
R45	3.24E+08	1.46	48.80	1.37E-09	3.32E+01	1.67E+02	5.76
R46	3.58E+08	1.45	48.98	1.22E-09	3.72E+01	1.63E+02	5.92
R47	2.38E+08	1.45	48.88	8.79E-10	5.18E+01	1.11E+02	8.66
R48	3.38E+08	1.46	48.81	1.39E-09	3.28E+01	1.70E+02	5.67
R49	1.96E+08	1.45	48.84	7.70E-10	5.91E+01	9.54E+01	1.01E+01
R50	1.57E+08	1.52	48.44	1.44E-09	3.16E+01	1.53E+02	6.31
R51	1.95E+08	1.53	48.42	1.90E-09	2.40E+01	2.00E+02	4.82
R52	2.19E+08	1.53	48.40	2.21E-09	2.06E+01	2.32E+02	4.15
R53	2.79E+08	1.54	48.41	2.86E-09	1.59E+01	3.01E+02	3.19
R54	2.55E+08	1.54	48.40	2.70E-09	1.69E+01	2.83E+02	3.40
R55	1.74E+08	1.54	48.40	1.80E-09	2.52E+01	1.89E+02	5.09

Table S4. Cartesian coordinates for all transition states.

TS1

	Cartesian Coordinates (Å)		
**At.**	**X**	**Y**	**Z**

**H**	-0.87120380	1.03786216	0.15049322
**O**	0.19455513	-1.01448602	-0.02790159
**O**	0.41267746	1.20540094	0.19351478
**C**	2.39860924	-0.10759710	0.07443852
**H**	2.80576776	0.44813158	-0.77371933
**H**	2.80505139	0.33821869	0.98507214
**H**	2.68640902	-1.15576575	0.00672544
**C**	0.89302749	0.03254505	0.08078426
**C**	-2.12201091	0.58718176	0.07785290
**H**	-2.55566104	0.90746655	1.02182306
**H**	-2.51522359	1.08197709	-0.80656047
**C**	-1.78077930	-0.76863893	-0.04371950
**H**	-1.83078273	-1.43588787	0.80671193
**H**	-1.79343620	-1.26440815	-1.00551531

TS2

Cartesian Coordinates (Å)			
**At.**	**X**	**Y**	**Z**
**H**	0.94665007	0.95748480	-0.19960668
**O**	-0.20514360	-1.04177598	-0.04586340
**O**	-0.36270344	1.17594621	-0.30981322
**C**	-2.37345342	-0.05219892	0.07111779
**H**	-2.63339309	0.36004960	1.05010444
**H**	-2.86714915	0.55821207	-0.68713145
**H**	-2.71196474	-1.08599081	0.01267670
**C**	-0.87355092	0.02869627	-0.11238838
**C**	2.16050973	0.53379303	0.02030333
**H**	2.64841530	0.89055116	-0.88803648
**C**	1.80114180	-0.82266560	-0.00148016
**H**	1.85075040	-1.39414919	-0.91849138
**H**	1.77316423	-1.41511178	0.90492301
**C**	2.61438318	1.18948987	1.31881286

**H**	2.47238611	2.27242600	1.28261211
**H**	2.03613774	0.81492191	2.16847953
**H**	3.67282052	1.00232166	1.52778176

TS3

Cartesian Coordinates (Å)			
**At.**	**X**	**Y**	**Z**
**H**	0.96629435	0.85599785	-0.18466724
**O**	-0.31511456	-1.06385451	-0.03207535
**O**	-0.32439216	1.15819871	-0.30905120
**C**	-2.41752015	0.06345832	0.02999303
**H**	-2.67535206	0.53364962	0.98291885
**H**	-2.85456831	0.66898112	-0.76623928
**H**	-2.81699165	-0.94963597	0.00414694
**C**	-0.91166666	0.04700112	-0.11794704
**C**	2.14711908	0.35140174	0.04328194
**H**	2.66554537	0.66055232	-0.86682555
**C**	1.69696503	-0.97864194	0.04121351
**H**	1.72114055	-1.57330145	-0.86200191
**H**	1.61795400	-1.54797072	0.95962644
**C**	2.64534574	0.99309731	1.33473097
**H**	2.49925542	2.07696779	1.27917573
**H**	2.01963954	0.64984950	2.16679662
**C**	4.11956846	0.69759590	1.64223531
**H**	4.44125358	1.19372180	2.56261951
**H**	4.29087460	-0.37672593	1.76122955
**H**	4.76665151	1.04665774	0.83183992

TS4

Cartesian Coordinates (Å)			
**At.**	**X**	**Y**	**Z**
**H**	1.04283487	0.66136238	-0.32873229

**O**	-0.37889305	-1.07930036	0.22084537
**O**	-0.22254859	1.02533652	-0.52736086
**C**	-2.38697395	0.19764328	0.04334830
**H**	-2.86552364	-0.76539745	0.21711637
**H**	-2.58973733	0.86258068	0.88747869
**H**	-2.78911050	0.67056320	-0.85446498
**C**	-0.88993831	0.03083999	-0.10107823
**C**	2.18322108	0.13027477	0.01388174
**H**	2.71639204	0.19789986	-0.93669297
**C**	1.63483654	-1.13219413	0.29181942
**H**	1.60816152	-1.90614891	-0.46332003
**H**	1.51934666	-1.48443281	1.30982117
**C**	2.73708517	0.99305481	1.14261090
**H**	2.68422724	2.04891503	0.85291835
**H**	2.08910289	0.89237736	2.02233652
**C**	4.18395553	0.65463486	1.53463732
**H**	4.23431724	-0.39513304	1.84829949
**H**	4.82257717	0.73614869	0.64705321
**C**	4.73237910	1.55294627	2.64617614
**H**	5.76260252	1.28978809	2.90058042
**H**	4.72318295	2.60479803	2.34415321
**H**	4.13250562	1.46644355	3.55757414

TS5

Cartesian Coordinates (Å)			
**At.**	**X**	**Y**	**Z**
**H**	1.00863248	0.76040180	-0.26610470
**O**	-0.40973594	-1.04109393	0.04494859
**O**	-0.25925893	1.14849444	-0.39918127
**C**	-2.42026963	0.24398991	0.05665034
**H**	-2.62080247	0.77326588	0.99235430
**H**	-2.82556361	0.84662833	-0.75815708

**H**	-2.89757293	-0.73483243	0.08491374
**C**	-0.92347974	0.10241203	-0.11538089
**C**	2.15199066	0.18759118	-0.01466253
**H**	2.67474565	0.38812790	-0.95218009
**C**	1.60545848	-1.10158945	0.08954665
**H**	1.57025690	-1.76240855	-0.76604136
**H**	1.49949580	-1.59252460	1.04949429
**C**	2.71926792	0.88453618	1.21767611
**H**	2.65809748	1.97029396	1.08085540
**H**	2.08368495	0.65627364	2.08231995
**C**	4.17258319	0.50104620	1.53639724
**H**	4.23239344	-0.58314159	1.69841355
**H**	4.79939996	0.71049036	0.66008843
**C**	4.74558026	1.23140819	2.75572118
**H**	4.68115643	2.31349605	2.58962641
**H**	4.11783299	1.02117439	3.63023346
**C**	6.19562835	0.84793262	3.06450907
**H**	6.57365159	1.38554006	3.93828677
**H**	6.28678179	-0.22342680	3.26908819
**H**	6.85404912	1.07991497	2.22158634

TS6

Cartesian Coordinates (Å)

At.	X	Y	Z
**H**	1.01781778	0.73523953	-0.29445009
**O**	-0.43136620	-1.02785432	0.08919001
**O**	-0.24433824	1.14683522	-0.41040302
**C**	-2.41244769	0.30209330	0.11995701
**H**	-2.57765314	0.86467977	1.04301666
**H**	-2.82598566	0.88772108	-0.70311490
**H**	-2.90927003	-0.66477446	0.19082789
**C**	-0.92392875	0.12283105	-0.08540314

**C**	2.15310671	0.14464625	-0.04778314
**H**	2.66384209	0.31107014	-0.99852497
**C**	1.58243807	-1.12997315	0.09768198
**H**	1.51812545	-1.81024850	-0.74080284
**H**	1.48396741	-1.59542040	1.07102646
**C**	2.75441616	0.86037169	1.15736219
**H**	2.71404231	1.94330227	0.99338563
**H**	2.12759170	0.66815508	2.03701574
**C**	4.20418691	0.45401492	1.46463467
**H**	4.24278493	-0.62670607	1.65317002
**H**	4.82125996	0.62777509	0.57388422
**C**	4.80911887	1.20321105	2.65649057
**H**	4.76623888	2.28309350	2.46417744
**H**	4.18997879	1.02963051	3.54638083
**C**	6.25669011	0.80341941	2.96438849
**H**	6.29979458	-0.27547804	3.15681112
**H**	6.87535158	0.97796638	2.07580035
**C**	6.85238564	1.55767845	4.15643049
**H**	7.88378819	1.25012164	4.34911767
**H**	6.85550152	2.63769833	3.97926313
**H**	6.27556784	1.37490137	5.06847253

TS7

	Cartesian Coordinates (Å)		
**At.**	**X**	**Y**	**Z**
**H**	1.01225042	0.73885949	-0.30005965
**O**	-0.48302365	-0.98744015	0.07161162
**O**	-0.23834767	1.18227773	-0.42460181
**C**	-2.42958163	0.39254115	0.09601300
**H**	-2.58432850	0.95015389	1.02392901
**H**	-2.82337909	0.99688085	-0.72315470
**H**	-2.95220086	-0.56142335	0.15548956

**C**	-0.94530964	0.17562670	-0.10378078
**C**	2.13137052	0.11977530	-0.04982319
**H**	2.64993330	0.27593052	-0.99807812
**C**	1.52800712	-1.14039817	0.08947687
**H**	1.45016516	-1.81658738	-0.75116182
**H**	1.41320224	-1.60572517	1.06109476
**C**	2.74669293	0.81597328	1.15968800
**H**	2.73486109	1.90012661	0.99918521
**H**	2.11242945	0.63703610	2.03681078
**C**	4.18460927	0.37108608	1.46943683
**H**	4.19443071	-0.71073705	1.65526228
**H**	4.80799243	0.53074005	0.58046872
**C**	4.80665738	1.10130916	2.66454713
**H**	4.79164138	2.18218482	2.47429516
**H**	4.18099833	0.94134289	3.55225866
**C**	6.24258600	0.66235387	2.97261762
**H**	6.25846338	-0.41868330	3.16328369
**H**	6.86857099	0.82252087	2.08514237
**C**	6.86674314	1.39284975	4.16707228
**H**	6.85192527	2.47278811	3.97654579
**H**	6.24176123	1.23286648	5.05402256
**C**	8.30090659	0.94776517	4.46700900
**H**	8.71636093	1.48709246	5.32261115
**H**	8.34346782	-0.12164242	4.69634515
**H**	8.95815159	1.12755682	3.61044684

TS8

Cartesian Coordinates (Å)

**At.**	**X**	**Y**	**Z**
**H**	0.97747266	0.81475301	-0.25967364
**O**	-0.47688018	-0.96059020	0.03869399
**O**	-0.28367240	1.23922571	-0.33314451

**C**	-2.45383568	0.37158991	0.14794827
**H**	-2.87100721	0.97302614	-0.66203051
**H**	-2.94881055	-0.59741508	0.19900513
**H**	-2.61713056	0.91605150	1.08196803
**C**	-0.96578765	0.19991241	-0.06733717
**C**	2.11353289	0.20406141	-0.07188550
**H**	2.60700179	0.42449019	-1.02076262
**C**	1.53680187	-1.07365798	0.00771609
**H**	1.45308553	-1.70273814	-0.86821939
**H**	1.45201161	-1.59537160	0.95343021
**C**	2.74439845	0.84116684	1.16208130
**H**	2.71116119	1.93233639	1.06428965
**H**	2.13296686	0.60137680	2.04089226
**C**	4.19602903	0.40462060	1.41488403
**H**	4.22752111	-0.68583040	1.53726139
**H**	4.79680765	0.62613501	0.52358749
**C**	4.83280133	1.07518499	2.63699952
**H**	4.79716475	2.16482764	2.51011153
**H**	4.22921670	0.85409869	3.52685447
**C**	6.28184059	0.64265976	2.88892133
**H**	6.31724350	-0.44718883	3.01533187
**H**	6.88491709	0.86425796	1.99894035
**C**	6.91986060	1.31315454	4.11071987
**H**	6.88583471	2.40316191	3.98476749
**H**	6.31706524	1.09245789	5.00131880
**C**	8.36862515	0.88068289	4.36382223
**H**	8.40279405	-0.20825772	4.49038221
**H**	8.97122614	1.10152315	3.47441337
**C**	8.99775973	1.55553665	5.58600325
**H**	10.02908781	1.22578685	5.73843244
**H**	9.01052035	2.64422420	5.47382500

**H**	8.43738549	1.32474848	6.49745694

TS9

Cartesian Coordinates (Å)			
**At.**	**X**	**Y**	**Z**
**H**	-0.26601060	1.35652152	-0.18116742
**O**	0.35382443	-0.84768307	0.25625901
**O**	1.03179408	1.16078453	-0.45938114
**C**	2.70350532	-0.45670952	0.07022540
**H**	3.27326585	-0.09741392	-0.78831980
**H**	3.13109410	-0.00063494	0.96789099
**H**	2.77354813	-1.54091885	0.15273026
**C**	1.25757579	-0.02432552	-0.05764221
**C**	-1.50164158	1.31628286	0.19731334
**H**	-1.53452653	1.99565567	1.04592419
**H**	-2.04329858	1.65477535	-0.68471369
**C**	-1.57009826	-0.06070830	0.48359780
**H**	-1.44987767	-0.38541078	1.51047801
**C**	-2.19595142	-1.04736169	-0.43900765
**H**	-1.78113803	-2.04440721	-0.29887987
**H**	-3.27017680	-1.08193904	-0.21262466
**H**	-2.08388844	-0.74450715	-1.48168258

TS10

Cartesian Coordinates (Å)			
**At.**	**X**	**Y**	**Z**
**H**	-0.33212824	-0.73771786	0.72900378
**O**	0.81689679	0.87098015	-0.49381637
**O**	1.02618068	-0.84426359	0.92423072
**C**	2.97072991	-0.14134455	-0.26990236
**H**	3.55011450	-0.34217870	0.63273689
**H**	3.10567278	-0.98924964	-0.94787357
**H**	3.32361697	0.76569053	-0.76004575

**C**	1.50100452	-0.02128680	0.08046827
**C**	-1.52895314	-0.56530295	0.33585474
**H**	-2.03725749	-0.43578368	1.29440009
**C**	-1.28058343	0.64061529	-0.34947212
**H**	-1.20666612	0.62038997	-1.43149735
**C**	-1.49406291	1.97851221	0.26567629
**H**	-0.84461676	2.72970456	-0.18123070
**H**	-2.53807052	2.26929436	0.08520117
**H**	-1.33705817	1.95129830	1.34554308
**C**	-1.90657738	-1.82119602	-0.44235014
**H**	-1.30859710	-1.91037631	-1.35369238
**H**	-1.72351855	-2.71744364	0.15467698
**H**	-2.96212664	-1.82534167	-0.73291134

TS11

Cartesian Coordinates (Å)			
**At.**	**X**	**Y**	**Z**
**H**	0.93167426	0.95070397	0.07361608
**O**	-0.24496210	-1.02317738	0.03894644
**O**	-0.40650023	1.20774954	0.12777564
**C**	-2.42511137	-0.05519527	-0.03609592
**H**	-2.87861662	0.56481264	0.73969276
**H**	-2.75021092	0.33616076	-1.00385562
**H**	-2.74680089	-1.09103340	0.06532201
**C**	-0.91725699	0.04790530	0.05377871
**C**	2.15006928	0.55606430	0.00781155
**C**	1.77569754	-0.79967616	-0.00808258
**H**	1.74633588	-1.36340088	-0.93162089
**H**	1.80182323	-1.39776700	0.89354517
**C**	2.66696207	1.17526336	-1.29085112
**H**	2.16159985	0.75435151	-2.16383813
**H**	2.49631431	2.25524093	-1.30147510

**H**	3.74413420	1.01207903	-1.41197845
**C**	2.76689349	1.11621429	1.29022002
**H**	2.32612494	0.65784507	2.17909719
**H**	3.84923465	0.94597832	1.32194656
**H**	2.60259676	2.19488164	1.36104570

TS12

Cartesian Coordinates (Å)			
**At.**	**X**	**Y**	**Z**
**H**	0.92576796	0.92775937	0.12248315
**O**	-0.26053696	-1.04054506	0.00979791
**O**	-0.41176929	1.18412760	0.20382619
**C**	-2.43217588	-0.05514954	-0.08114529
**H**	-2.90634905	0.57391514	0.67437970
**H**	-2.71930019	0.33187535	-1.06280959
**H**	-2.76736155	-1.08749517	0.01265546
**C**	-0.92716385	0.03293530	0.05900173
**C**	2.14023326	0.53111682	0.01758451
**C**	1.76162887	-0.82296897	-0.03343331
**H**	1.72736833	-1.36532409	-0.96953106
**H**	1.78886652	-1.44319040	0.85336258
**C**	2.62553592	1.18789991	-1.27640164
**H**	2.05305920	0.83218922	-2.13693799
**H**	2.50239962	2.27339503	-1.22847338
**H**	3.68315624	0.98411254	-1.47342030
**C**	2.76189194	1.04676220	1.32327170
**H**	2.20896910	0.62348468	2.16876685
**H**	2.61169816	2.13043649	1.38003448
**C**	4.25659250	0.73407407	1.48374420
**H**	4.85047478	1.17805674	0.68051465
**H**	4.63826512	1.12787814	2.42997440
**H**	4.43775158	-0.34534467	1.47375538

TS13

Cartesian Coordinates (Å)			
**At.**	**X**	**Y**	**Z**
**H**	-0.19098159	-0.62403833	0.54789470
**O**	1.21905825	0.78339430	-0.62945681
**O**	1.14222925	-0.77383965	0.97219629
**C**	3.27594388	-0.22644435	0.04916369
**H**	3.69313776	-0.36978402	1.04712947
**H**	3.49349652	-1.12465705	-0.53640597
**H**	3.73287246	0.63355113	-0.44016715
**C**	1.77219924	-0.05211698	0.13999633
**C**	-1.32867141	-0.43094759	0.05800260
**C**	-0.89673942	0.60720640	-0.80374972
**H**	-0.64516626	0.33257240	-1.82194045
**C**	-1.67832467	-1.76758039	-0.60371824
**H**	-1.01457851	-1.98209042	-1.44523734
**H**	-1.57979277	-2.58870865	0.11073462
**H**	-2.70936427	-1.77457958	-0.97555272
**C**	-2.18706942	-0.08612971	1.27629937
**H**	-1.78619820	0.75654561	1.84267527
**H**	-2.22357644	-0.94093444	1.95564370
**H**	-3.21832568	0.15477414	0.99318521
**C**	-1.10536966	2.06066995	-0.57009661
**H**	-1.00376708	2.32853568	0.48137108
**H**	-0.41374911	2.65645949	-1.16280562
**H**	-2.13326329	2.30514205	-0.87516175

TS14

Cartesian Coordinates (Å)			
**At.**	**X**	**Y**	**Z**
**H**	0.33657513	-0.27444433	-0.66570856

**O**	-1.49929684	0.65035609	0.42559831
**O**	-0.80459653	-1.01959384	-0.88549861
**C**	-2.87141917	-1.30251843	0.27884303
**H**	-3.25091293	-1.81972523	-0.60386477
**H**	-2.58948375	-2.06161585	1.01454227
**H**	-3.63971816	-0.66007935	0.70840489
**C**	-1.64390528	-0.49283584	-0.08855423
**C**	1.29308180	0.44626575	-0.24174709
**C**	0.47688139	1.42647151	0.35540952
**H**	0.35391811	1.41793974	1.43308414
**C**	0.07913620	2.68150542	-0.33700162
**H**	0.02090980	2.54088508	-1.41780685
**H**	-0.87237072	3.05634072	0.03710590
**H**	0.85253781	3.43588845	-0.13707915
**H**	1.74486993	0.75601424	-1.18851971
**C**	2.16142663	-0.46493567	0.62664933
**H**	1.59339270	-0.74898516	1.51979523
**H**	3.04179887	0.08321126	0.98421258
**C**	2.61382801	-1.72891321	-0.11136632
**H**	3.20020720	-1.47662358	-1.00040006
**H**	3.23815080	-2.35729222	0.52913652
**H**	1.75298940	-2.31831557	-0.43623469

TS15

Cartesian Coordinates (Å)			
**At.**	**X**	**Y**	**Z**
**H**	1.04276465	0.93544196	0.25478931
**O**	-0.20679555	-0.98938013	0.07780996
**O**	-0.28664043	1.22683212	0.36204187
**C**	-2.31937253	0.09237183	-0.17798683
**H**	-2.83416637	0.77617966	0.49872286
**H**	-2.48246752	0.44692415	-1.19994299

**H**	-2.71803868	-0.91727243	-0.08360065
**C**	-0.83397077	0.10748832	0.11411073
**C**	2.24402527	0.51301012	0.09650684
**C**	1.82653245	-0.82730132	0.00912948
**H**	1.75218645	-1.33253699	-0.94539287
**C**	2.73018854	1.18752309	-1.19601241
**H**	2.22828694	0.71537821	-2.04757885
**H**	3.80299494	0.98904383	-1.32356683
**C**	2.48736378	2.70081672	-1.25324708
**H**	3.02887869	3.22756354	-0.46310021
**H**	2.82634472	3.10982759	-2.20878367
**H**	1.42486683	2.93121616	-1.14162676
**H**	1.85529183	-1.47898823	0.87265802
**C**	2.93155733	0.95377452	1.39199990
**H**	4.01203263	0.77407293	1.34876715
**H**	2.78209101	2.01884634	1.58192697
**H**	2.53304781	0.41216929	2.25337584

TS16

Cartesian Coordinates (Å)

At.	X	Y	Z
**H**	1.02137769	1.05226555	0.14596757
**O**	-0.20456980	-0.87709404	0.43449965
**O**	-0.31498804	1.33787516	0.15036564
**C**	-2.31957360	0.06976560	-0.13946095
**H**	-2.86073373	0.89208193	0.33119545
**H**	-2.45647149	0.15507513	-1.22144261
**H**	-2.71071540	-0.89088880	0.19428334
**C**	-0.84344254	0.18288363	0.17771884
**C**	2.22873865	0.62197728	0.12077106
**C**	1.82936885	-0.70238172	0.37723934
**H**	1.78791412	-1.44355270	-0.41071400

**C**	2.71896231	0.94412047	-1.30144719
**H**	2.18835081	0.29124877	-2.00293847
**H**	3.78144643	0.68777573	-1.39679709
**C**	2.51060514	2.40227496	-1.72766038
**H**	3.08288318	3.09342993	-1.10298769
**H**	2.83595367	2.55190869	-2.76062901
**H**	1.45681825	2.68389998	-1.65860175
**H**	1.84017910	-1.10313265	1.38274572
**C**	2.86548898	1.39565630	1.28677165
**H**	2.70796253	2.46844824	1.13997275
**H**	2.32657827	1.14247272	2.20589447
**C**	4.36405031	1.12543572	1.48235271
**H**	4.94497128	1.40983453	0.60096220
**H**	4.75504162	1.69482963	2.33029163
**H**	4.55280644	0.06479150	1.67564713

TS17

Cartesian Coordinates (Å)			
**At.**	**X**	**Y**	**Z**
**H**	1.10145750	0.63945056	0.34489327
**O**	-0.28861370	-1.09393379	-0.30495691
**O**	-0.16974052	0.94248119	0.61284227
**C**	-2.30824049	0.16437862	-0.11256096
**H**	-2.78478255	-0.79229468	-0.32362216
**H**	-2.74134548	0.62318925	0.77779480
**H**	-2.47656429	0.84378683	-0.95310296
**C**	-0.81849221	-0.01487684	0.08217632
**C**	2.24275394	0.19252835	-0.09849800
**C**	1.73405376	-1.06991895	-0.43761130
**H**	1.58804277	-1.36227703	-1.47049911
**C**	2.70872333	1.16049890	-1.19003676
**H**	2.00692269	1.06775039	-2.02962712

**C**	2.66055719	2.61414462	-0.69774786
**H**	3.33344041	2.75695534	0.15487612
**H**	2.97335183	3.30622475	-1.48466944
**H**	1.65288203	2.89008200	-0.37875114
**H**	1.76066729	-1.88868668	0.26859924
**H**	2.82235589	0.22114677	0.82791438
**C**	4.11222618	0.79997849	-1.70423885
**H**	4.84818690	0.86285626	-0.89566194
**H**	4.14239111	-0.21865155	-2.10200524
**H**	4.43076876	1.48218821	-2.49850762

TS18

Cartesian Coordinates (Å)			
**At.**	**X**	**Y**	**Z**
**H**	0.89930357	0.98567747	0.29393693
**O**	-0.12632746	-1.06447716	-0.04138054
**O**	-0.37125473	1.09230806	0.50753685
**C**	-2.34885153	-0.24218150	0.22045297
**H**	-2.65619008	-0.14490553	-0.82819901
**H**	-2.55481204	-1.27761904	0.50238548
**C**	-0.84014166	-0.05995454	0.23788618
**C**	2.14379538	0.61095173	-0.00577264
**H**	2.39957974	1.22959351	-0.86200030
**H**	2.68596680	0.83484213	0.90959472
**C**	1.83095470	-0.73380958	-0.25259649
**H**	1.73810609	-1.11191587	-1.26223777
**H**	2.00183667	-1.49336722	0.49873565
**C**	-3.11273461	0.75506559	1.09255481
**H**	-2.86797548	1.78055553	0.81386488
**H**	-4.19031109	0.61196442	0.98269310
**H**	-2.86094487	0.63027217	2.14854553

TS19

Cartesian Coordinates (Å)

At.	X	Y	Z
**H**	0.67228537	1.33553657	0.10401009
**O**	0.19283736	-0.90845987	-0.29371506
**O**	-0.62972992	1.08618390	0.28947747
**C**	-2.17841148	-0.63455986	-0.35954186
**H**	-2.32242816	-0.58966070	-1.44627892
**H**	-2.18949421	-1.69603110	-0.09854450
**C**	-0.77123972	-0.11842155	-0.09435342
**C**	1.93405036	1.35259018	-0.18743330
**H**	1.98898119	2.00527190	-1.05555751
**H**	2.39128912	1.74950734	0.71772444
**C**	2.09316805	-0.02657902	-0.41741611
**H**	2.06005674	-0.39186160	-1.43713658
**C**	-3.28314105	0.14246288	0.35675600
**H**	-3.23651658	1.20247756	0.10447812
**H**	-4.26733809	-0.24029928	0.07517196
**H**	-3.18514984	0.05841505	1.44190109
**C**	2.69738487	-0.94878453	0.58260498
**H**	2.34033999	-1.96909707	0.45148413
**H**	3.78545644	-0.93825919	0.43244844
**H**	2.49759960	-0.61643163	1.60292061

TS20

Cartesian Coordinates (Å)

At.	X	Y	Z
**H**	-0.63148805	-0.74130613	0.66625886
**O**	0.20950972	1.04885464	-0.55101216
**O**	0.73840781	-0.68323734	0.75557591
**C**	2.47938865	0.30476619	-0.57969015
**H**	2.49239540	-0.17128166	-1.56807965
**H**	2.70353001	1.36075076	-0.75246051

**C**	1.04175921	0.22277899	-0.08306309
**C**	-1.86896171	-0.71809860	0.36780250
**H**	-2.31373343	-0.69312065	1.36553174
**C**	-1.83187403	0.53345955	-0.27684597
**H**	-1.83176448	0.56247687	-1.36118535
**C**	3.50561694	-0.35330085	0.34267299
**H**	3.24803155	-1.39729165	0.52507786
**H**	4.50418070	-0.31211403	-0.09985085
**H**	3.54412222	0.14906545	1.31239908
**C**	-2.16908938	1.80868894	0.41101576
**H**	-1.65330127	2.65330218	-0.04317089
**H**	-3.25174113	1.96810959	0.31263587
**H**	-1.93374793	1.76273802	1.47588892
**C**	-2.13403716	-1.98545956	-0.43764407
**H**	-1.60191792	-1.95964042	-1.39273823
**H**	-1.78738480	-2.86893193	0.10321605
**H**	-3.19890106	-2.12320848	-0.65133463

TS21

Cartesian Coordinates (Å)			
**At.**	**X**	**Y**	**Z**
**H**	0.97011050	0.86037742	0.28306336
**O**	-0.17488552	-1.11847787	0.07401520
**O**	-0.35392994	1.06370951	0.52651053
**C**	-2.37421729	-0.20937009	0.22674589
**H**	-2.63707082	-0.24040971	-0.83777395
**H**	-2.63013354	-1.19375284	0.62748613
**C**	-0.85944128	-0.07663454	0.28823875
**C**	2.17880873	0.52785550	0.00534815
**C**	1.82891341	-0.82245864	-0.16911801
**H**	1.69602728	-1.24637470	-1.15609788
**C**	-3.13474064	0.91256246	0.93361611

**H**	-2.84549156	1.88671473	0.53753770
**H**	-4.21229280	0.78830075	0.80073733
**H**	-2.92394512	0.91817051	2.00572022
**C**	2.51653662	1.35216469	-1.23666214
**H**	1.91671676	1.04428373	-2.09695071
**H**	2.32240389	2.41420820	-1.06364305
**H**	3.57375027	1.25204135	-1.50836432
**H**	1.96985086	-1.54357220	0.62530145
**C**	2.93798773	0.91603989	1.27502283
**H**	2.61806672	0.31638718	2.13084439
**H**	4.01879818	0.78224073	1.15162585
**H**	2.76417822	1.96599469	1.52579654

TS22

Cartesian Coordinates (Å)			
**At.**	**X**	**Y**	**Z**
**H**	0.94465549	0.85128770	0.34538256
**O**	-0.19903058	-1.12675554	0.09488104
**O**	-0.38313274	1.04880969	0.57464001
**C**	-2.39750938	-0.20498731	0.17052568
**H**	-2.61676734	-0.20456166	-0.90432928
**H**	-2.67541652	-1.19808254	0.53318877
**C**	-0.88576855	-0.08419153	0.29747654
**C**	2.15464951	0.52134501	0.07208332
**C**	1.81006376	-0.83025395	-0.10600574
**H**	1.69764322	-1.26049760	-1.09281135
**C**	-3.17981045	0.90256462	0.87646157
**H**	-2.86880546	1.88503817	0.51936340
**H**	-4.25175431	0.78952657	0.69651293
**H**	-3.01303951	0.87738469	1.95604206
**C**	2.48768265	1.34696964	-1.17207801
**H**	1.84511028	1.07029964	-2.01191866

**H**	2.33519130	2.41283367	-0.98126213
**H**	3.52670702	1.21424928	-1.49094123
**H**	1.93625985	-1.54767971	0.69453449
**C**	2.88663737	0.90480226	1.36612692
**H**	2.43220503	0.36284529	2.20244057
**H**	2.71155741	1.96732492	1.56740894
**C**	4.39830283	0.63626697	1.34534009
**H**	4.89676177	1.19494946	0.54884636
**H**	4.85995132	0.93183816	2.29170265
**H**	4.60865769	-0.42632505	1.18838918

TS23

	Cartesian Coordinates (Å)		
**At.**	**X**	**Y**	**Z**
**H**	-0.47358758	-0.71854395	0.23673451
**O**	0.61562857	1.22053994	-0.39531664
**O**	0.90736699	-0.81427798	0.47687433
**C**	2.84000481	0.35244094	-0.35866347
**H**	2.95298762	0.20027915	-1.43912049
**H**	3.13401004	1.38905660	-0.17192633
**C**	1.34858787	0.24656411	-0.06310827
**C**	-1.67794360	-0.54091059	-0.06823044
**C**	-1.47809262	0.79534102	-0.49132815
**H**	-1.31341738	0.96005513	-1.55016789
**C**	3.71204117	-0.63507591	0.41607383
**H**	3.38952186	-1.66123535	0.23512552
**H**	4.75955588	-0.54082697	0.11818048
**H**	3.64828428	-0.45772605	1.49238843
**C**	-1.93639172	-1.57648208	-1.16692648
**H**	-1.35772611	-1.35614565	-2.06762161
**H**	-1.65182531	-2.57610299	-0.82904129
**H**	-2.99595348	-1.61053649	-1.44519918

**C**	-2.41921588	-0.81184222	1.24207615
**H**	-2.06222801	-0.18334902	2.06005375
**H**	-2.26629442	-1.85036299	1.54477473
**H**	-3.49897669	-0.65366018	1.13868067
**C**	-1.82557440	2.00810925	0.29501695
**H**	-1.63406855	1.87874186	1.36000115
**H**	-1.28126013	2.87854199	-0.06628909
**H**	-2.90343346	2.18940829	0.17295888

TS24

Cartesian Coordinates (Å)			
**At.**	**X**	**Y**	**Z**
**H**	-0.56632467	-0.29680581	0.61396471
**O**	0.71615974	1.29381546	-0.49632018
**O**	0.77762596	-0.57438711	0.72330465
**C**	2.71701776	-0.01095473	-0.58536815
**H**	2.60497308	-0.49402428	-1.56422999
**H**	3.18660325	0.95713086	-0.77737177
**C**	1.30732517	0.26102201	-0.07704350
**C**	-1.75370747	0.02951245	0.29014337
**C**	-1.40465967	1.26786664	-0.28066078
**H**	-1.36011779	1.35090860	-1.36128510
**C**	3.56414662	-0.88325302	0.34187192
**H**	3.06194805	-1.82932082	0.54725930
**H**	4.53687167	-1.09487226	-0.10934784
**H**	3.73706246	-0.38777570	1.30059106
**C**	-2.28225992	-1.10315650	-0.59080993
**H**	-1.71161117	-1.12003937	-1.52629236
**H**	-3.32453077	-0.90617119	-0.87056221
**C**	-1.45074869	2.55020318	0.47148171
**H**	-1.27054045	2.39602360	1.53687200
**H**	-0.73255687	3.26727376	0.07680503

**H**	-2.45962046	2.97059156	0.35830061
**C**	-2.18833998	-2.47364827	0.08734944
**H**	-2.76486994	-2.49267096	1.01749214
**H**	-2.58049172	-3.26175792	-0.56072405
**H**	-1.15156555	-2.71596842	0.33294525
**H**	-2.21578887	0.10545803	1.27863468

TS25

Cartesian Coordinates (Å)			
**At.**	**X**	**Y**	**Z**
**H**	1.09596525	0.82193376	0.36518139
**O**	-0.13245192	-1.10776393	0.13016864
**O**	-0.21793772	1.06797230	0.62730104
**C**	-2.28085638	-0.06739514	0.12745790
**H**	-2.45805306	0.03667771	-0.95035113
**H**	-2.62069036	-1.07092285	0.39538205
**C**	-0.77166642	-0.03341700	0.31719476
**C**	2.28538124	0.45310707	0.04606958
**C**	1.88568870	-0.88337125	-0.12811693
**H**	1.72066092	-1.29785899	-1.11442283
**C**	-3.03473169	1.01798211	0.89692801
**H**	-2.65719536	2.00812267	0.63915867
**H**	-4.10229102	0.98094927	0.66659515
**H**	-2.91716605	0.89076471	1.97595269
**C**	2.62360554	1.26064762	-1.21684105
**H**	2.04540227	0.85775382	-2.05547764
**H**	3.68093670	1.10414346	-1.47007949
**C**	2.35083028	2.76554322	-1.10282897
**H**	2.96102286	3.22944186	-0.32342405
**H**	2.58401959	3.27167002	-2.04340015
**H**	1.30151896	2.95643851	-0.86438301
**H**	2.01165518	-1.61225947	0.66173826

**C**	3.09107786	0.79049129	1.30388397
**H**	4.16500554	0.64841179	1.13647730
**H**	2.93992953	1.82789145	1.61006127
**H**	2.79334087	0.15804743	2.14377468

TS26

Cartesian Coordinates (Å)

At.	X	Y	Z
**H**	1.06104554	0.91122510	0.36377009
**O**	-0.09584827	-1.07011085	0.17092813
**O**	-0.26306963	1.11164233	0.61765602
**C**	-2.27752656	-0.10283604	0.10604142
**H**	-2.43823425	-0.01895232	-0.97613804
**H**	-2.58812363	-1.11361844	0.38193299
**C**	-0.77404254	-0.01436180	0.32250398
**C**	2.26298728	0.57730257	0.05738567
**C**	1.91668722	-0.77900278	-0.07480417
**H**	1.77861543	-1.23586461	-1.04645487
**C**	-3.08190383	0.96655245	0.84634268
**H**	-2.73235854	1.96529264	0.58233092
**H**	-4.14299072	0.89097355	0.59632763
**H**	-2.98121129	0.85715900	1.92904845
**C**	2.55968254	1.35595795	-1.23573404
**H**	1.95076661	0.92957064	-2.04027098
**H**	3.60389409	1.19882137	-1.53339605
**C**	2.28105530	2.86122040	-1.14762096
**H**	2.92014106	3.34961734	-0.40703229
**H**	2.46991543	3.34478803	-2.10977777
**H**	1.24148663	3.05196127	-0.86938343
**H**	2.06110269	-1.47402011	0.74227405
**C**	3.03378280	0.98013446	1.32502285
**H**	2.84316486	2.03452568	1.54677183

**H**	2.62234527	0.42219330	2.17275319
**C**	4.54883121	0.74486186	1.24516842
**H**	5.00612787	1.32254765	0.43747167
**H**	5.03844525	1.04033715	2.17738921
**H**	4.77623451	-0.31091607	1.06749378

TS27

Cartesian Coordinates (Å)			
**At.**	**X**	**Y**	**Z**
**H**	1.13652174	0.67745261	0.41422919
**O**	-0.17398122	-1.19578260	0.06180197
**O**	-0.13650536	0.94836988	0.68975103
**C**	-2.25671090	-0.03009956	0.11005288
**H**	-2.41680109	0.17978384	-0.95497817
**H**	-2.65595979	-1.03171910	0.28701102
**C**	-0.75085028	-0.09984269	0.30922063
**C**	2.28840390	0.23827072	-0.01786121
**C**	1.84220649	-1.08239579	-0.16664181
**H**	1.67243822	-1.51572331	-1.14491836
**C**	-2.95397554	1.02601753	0.96891141
**H**	-2.51740367	2.01112520	0.80086990
**H**	-4.01944309	1.07157904	0.73100219
**H**	-2.85486281	0.79764192	2.03302534
**C**	2.65607576	1.07744430	-1.24500540
**H**	1.92902890	0.83963736	-2.03295846
**C**	2.54521667	2.57875788	-0.94262038
**H**	3.24065861	2.86608191	-0.14641165
**H**	2.78897196	3.17647281	-1.82543364
**H**	1.53644239	2.84308207	-0.61740416
**H**	1.94078626	-1.79769178	0.63850873
**H**	2.89689789	0.42164455	0.87158199
**C**	4.05701138	0.72445166	-1.77170000

**H**	4.81875874	0.92970408	-1.01209720
**H**	4.12909449	-0.33456343	-2.03630660
**H**	4.30598200	1.31230211	-2.66062983

TS28

Cartesian Coordinates (Å)			
**At.**	**X**	**Y**	**Z**
**H**	0.87219555	1.03388809	0.12340021
**O**	-0.06842915	-1.08297209	0.05801676
**O**	-0.40396391	1.11616211	0.31492346
**C**	-2.32143361	-0.32172002	0.27397901
**H**	-2.71463312	-0.08632742	-0.72362142
**H**	-2.48326245	-1.39010755	0.43703017
**C**	-0.82355966	-0.08228728	0.21715399
**C**	2.13383249	0.67164611	-0.11149114
**H**	2.37799604	1.18513075	-1.03783425
**H**	2.65657907	1.03019207	0.77170979
**C**	1.87388494	-0.70485771	-0.18780793
**H**	1.80549691	-1.21007352	-1.14233142
**H**	2.06776574	-1.35703724	0.65353352
**C**	-3.04558388	0.52983913	1.32484694
**H**	-2.79241893	1.57977104	1.16042836
**H**	-2.66452745	0.27331019	2.31955474
**C**	-4.56365405	0.33834370	1.29045523
**H**	-5.05492739	0.94881453	2.05271281
**H**	-4.97674343	0.62535271	0.31831129
**H**	-4.83961535	-0.70506744	1.47203056

TS29

Cartesian Coordinates (Å)			
**At.**	**X**	**Y**	**Z**
**H**	0.88074767	0.98945344	0.04731989

**O**	-0.10116840	-1.10092753	0.00112581
**O**	-0.44043354	1.10549230	0.16291606
**C**	-2.34994415	-0.34222225	0.26883584
**H**	-2.77923198	-0.18055129	-0.72862189
**H**	-2.49621257	-1.39819051	0.50982544
**C**	-0.85600822	-0.09669961	0.14154994
**C**	2.14139478	0.65548528	-0.00832515
**H**	2.44598663	1.18827146	-0.91015516
**C**	1.87370039	-0.70944431	-0.19266140
**H**	1.81871612	-1.13614689	-1.18518173
**H**	2.02330460	-1.42675016	0.60496064
**C**	-3.04683382	0.57487134	1.28168459
**H**	-2.80556006	1.61223253	1.03849040
**H**	-2.63300521	0.38875281	2.27903462
**C**	-4.56401556	0.37496948	1.30899300
**H**	-5.03538228	1.03381099	2.04313598
**H**	-5.00929608	0.59310060	0.33313467
**H**	-4.82745455	-0.65487723	1.56964829
**C**	2.75684160	1.14972699	1.29546736
**H**	2.54135576	2.20923641	1.45438058
**H**	2.34853956	0.60402045	2.15100472
**H**	3.84495827	1.02838616	1.31043856

TS30

Cartesian Coordinates (Å)

At.	X	Y	Z
**H**	1.24737900	-1.32545992	-0.25730954
**O**	0.67714936	0.87590087	0.24884163
**O**	-0.02188961	-1.06885625	-0.60601147
**C**	-1.65717395	0.63982230	-0.22203287
**H**	-1.69140543	1.56155398	0.36420071
**H**	-1.86519224	0.91308019	-1.26459430

**C**	-0.23132767	0.11345783	-0.18386808
**C**	2.46117088	-1.35289116	0.18917975
**H**	2.41868653	-2.07392084	1.00226558
**C**	2.55984570	0.00440879	0.54934580
**H**	2.38555441	0.28264578	1.58202694
**C**	-2.70347361	-0.37536969	0.25653136
**H**	-2.50649049	-0.62696656	1.30485406
**H**	-2.57656162	-1.30056113	-0.31046965
**C**	-4.13653813	0.14286984	0.11389498
**H**	-4.86072575	-0.59558220	0.46845656
**H**	-4.28925822	1.06089152	0.69003315
**H**	-4.37400349	0.36534625	-0.93123506
**H**	3.04074401	-1.66837799	-0.67708221
**C**	3.27370584	1.01220946	-0.28178879
**H**	2.88278199	2.01511920	-0.11704164
**H**	4.33195925	0.99822564	0.01226249
**H**	3.21906306	0.76445412	-1.34345935

TS31

Cartesian Coordinates (Å)			
**At.**	**X**	**Y**	**Z**
**H**	1.15615998	-0.74276264	-0.67137071
**O**	0.29561744	1.01984514	0.57285092
**O**	-0.21476189	-0.69617434	-0.76181248
**C**	-1.97023140	0.25310438	0.57501994
**H**	-2.04339841	-0.43944647	1.42435291
**H**	-2.14722540	1.25538325	0.97453741
**C**	-0.52909020	0.19512100	0.08926638
**C**	2.39265275	-0.70956555	-0.37234725
**C**	2.34099603	0.53235961	0.29024209
**H**	2.34167791	0.54593937	1.37489967
**C**	-3.00779509	-0.12439825	-0.48788979

**H**	-2.74189164	-1.09785873	-0.90646389
**H**	-2.94936643	0.58973112	-1.31678042
**C**	-4.43491542	-0.15508714	0.06485782
**H**	-5.15512978	-0.41854586	-0.71450581
**H**	-4.53230859	-0.89158814	0.86895420
**H**	-4.72765637	0.81802778	0.47175977
**H**	2.83769518	-0.66489823	-1.36927870
**C**	2.66384969	1.82087544	-0.37966707
**H**	2.14016772	2.65363819	0.08723286
**H**	3.74494132	1.99005972	-0.28055606
**H**	2.42744131	1.78768156	-1.44479472
**C**	2.67313206	-1.98487142	0.41517174
**H**	2.14063682	-1.97908248	1.37038643
**H**	2.33723363	-2.86472285	-0.13824397
**H**	3.73956867	-2.11276453	0.62717877

TS32

	Cartesian Coordinates (Å)		
**At.**	**X**	**Y**	**Z**
**H**	0.97369905	0.86692848	0.24526911
**O**	-0.10530032	-1.15596903	0.11310301
**O**	-0.35546315	1.03370642	0.49017594
**C**	-2.33319202	-0.31317775	0.28955071
**H**	-2.66557576	-0.07620285	-0.72982885
**H**	-2.54503991	-1.37332338	0.45123537
**C**	-0.82382115	-0.13294984	0.30588971
**C**	2.19130887	0.56393581	-0.02594371
**C**	1.88451758	-0.80232311	-0.15327397
**H**	1.76097808	-1.26296314	-1.12493610
**C**	-3.07789170	0.57508021	1.29316410
**H**	-2.77155293	1.61214563	1.13775169
**H**	-2.76274359	0.31172945	2.30892509

**C**	-4.59871960	0.44720936	1.17688972
**H**	-5.10516428	1.08329558	1.90778639
**H**	-4.94592625	0.74373767	0.18199928
**H**	-4.92814269	-0.58241848	1.34826431
**C**	2.49721000	1.35583936	-1.29707655
**H**	1.90315960	1.00052113	-2.14296910
**H**	2.27060648	2.41656255	-1.15879335
**H**	3.55573559	1.27967412	-1.57151961
**H**	2.05438371	-1.49189537	0.66328260
**C**	2.94427388	1.01825391	1.22533206
**H**	2.64761910	0.43825114	2.10283171
**H**	4.02818643	0.91419131	1.10016102
**H**	2.73885459	2.07016157	1.44173013

TS33

	Cartesian Coordinates (Å)		
**At.**	**X**	**Y**	**Z**
**H**	0.95695828	0.83764479	0.33261450
**O**	-0.13664468	-1.17526495	0.13887189
**O**	-0.37269431	1.00401880	0.57803316
**C**	-2.35926555	-0.31075829	0.27277127
**H**	-2.65489601	-0.04919628	-0.75195988
**H**	-2.58888034	-1.37134242	0.40484604
**C**	-0.84906199	-0.15017851	0.34221205
**C**	2.17128879	0.53312114	0.05104901
**C**	1.85907198	-0.83019918	-0.09701109
**H**	1.74478005	-1.28168347	-1.07410089
**C**	-3.12667475	0.56555831	1.26960650
**H**	-2.80349316	1.60183645	1.14634412
**H**	-2.84851703	0.27762152	2.28947138
**C**	-4.64416609	0.45852752	1.10038957
**H**	-5.16745395	1.08549185	1.82730012

**H**	-4.95462520	0.77966169	0.10098918
**H**	-4.99086133	-0.57035651	1.23909728
**C**	2.46746577	1.34243196	-1.21308414
**H**	1.82059580	1.03421814	-2.03852091
**H**	2.29141243	2.40775803	-1.04031099
**H**	3.50506830	1.22890949	-1.54383459
**H**	2.01596894	-1.52920630	0.71441140
**C**	2.91064112	0.95930114	1.32737793
**H**	2.48123033	0.42228716	2.17997484
**H**	2.71153991	2.02078242	1.51115719
**C**	4.42823068	0.72828865	1.29090678
**H**	4.90184017	1.28412303	0.47742825
**H**	4.89482873	1.05308838	2.22520218
**H**	4.66331373	-0.33148369	1.15076888

TS34

Cartesian Coordinates (Å)			
**At.**	**X**	**Y**	**Z**
**H**	-0.94976867	-0.72845056	0.21186729
**O**	0.09146007	1.23199533	-0.43674591
**O**	0.43905746	-0.80307336	0.41348550
**C**	2.33080861	0.39372467	-0.46073959
**H**	2.46009194	-0.00804312	-1.47480719
**H**	2.57050292	1.45917898	-0.51783914
**C**	0.84955107	0.26905913	-0.12981356
**C**	-2.16451335	-0.56593421	-0.05676002
**C**	-1.99614755	0.77620251	-0.47569673
**H**	-1.86480485	0.95057322	-1.53767304
**C**	3.25905433	-0.34460589	0.50908800
**H**	2.92993507	-1.38349228	0.58634710
**H**	3.14721424	0.08460784	1.51092547
**C**	4.72821546	-0.28220746	0.08358655

**H**	5.36917046	-0.80907305	0.79577126
**H**	4.87585760	-0.74249110	-0.89871789
**H**	5.08297688	0.75141893	0.01976255
**C**	-2.44089597	-1.59702053	-1.15538565
**H**	-1.89153959	-1.36241417	-2.07072168
**H**	-2.13331234	-2.59508483	-0.83342080
**H**	-3.50752252	-1.64349594	-1.40327762
**C**	-2.86420394	-0.85603679	1.27221328
**H**	-2.49109923	-0.22955198	2.08454792
**H**	-2.69001290	-1.89489805	1.56199786
**H**	-3.94837299	-0.71036591	1.20156336
**C**	-2.33971166	1.97813467	0.32890432
**H**	-2.11550284	1.84475648	1.38700236
**H**	-1.82010080	2.85968145	-0.04176667
**H**	-3.42338695	2.14290589	0.23930270

TS35

Cartesian Coordinates (Å)			
**At.**	**X**	**Y**	**Z**
**H**	-1.02477184	-0.31096855	0.61551079
**O**	0.11139201	1.37804575	-0.50906980
**O**	0.33955365	-0.46252017	0.73237745
**C**	2.22657467	0.25983256	-0.56759569
**H**	2.19409440	-0.41867517	-1.43068052
**H**	2.55277389	1.23231346	-0.94596842
**C**	0.79433381	0.40959544	-0.07563157
**C**	-2.23475184	-0.09718858	0.28501381
**C**	-1.99738886	1.16232988	-0.29740600
**H**	-1.95758599	1.23860837	-1.37873027
**C**	3.19614290	-0.29225052	0.48351759
**H**	2.78676693	-1.22318775	0.88271787
**H**	3.24668890	0.40518206	1.32705849

**C**	4.60194301	-0.52616750	-0.07508852
**H**	5.27465402	-0.91145795	0.69596476
**H**	4.58658580	-1.25190825	-0.89457259
**H**	5.03776472	0.40003468	-0.46290724
**C**	-2.65520908	-1.28151293	-0.58621505
**H**	-2.08589029	-1.25223279	-1.52220761
**H**	-3.71146722	-1.18535123	-0.86632316
**C**	-2.16283986	2.44238866	0.44181713
**H**	-1.97170152	2.31590775	1.50895326
**H**	-1.51288503	3.21876013	0.04103524
**H**	-3.20585328	2.76699826	0.32308136
**H**	-2.70599407	-0.05358643	1.27113449
**C**	-2.43266148	-2.63173769	0.10259844
**H**	-3.00440781	-2.69750906	1.03357321
**H**	-2.74930007	-3.45834347	-0.53879807
**H**	-1.37756044	-2.77339922	0.34884063

TS36

Cartesian Coordinates (Å)			
**At.**	**X**	**Y**	**Z**
**H**	1.11311234	0.80011073	0.38344690
**O**	-0.03643711	-1.18244236	0.18969175
**O**	-0.20341510	0.99027358	0.67943290
**C**	-2.22859309	-0.23309261	0.25937063
**H**	-2.47359631	0.07660833	-0.76538262
**H**	-2.50492422	-1.28734798	0.34395836
**C**	-0.71748490	-0.13701039	0.39281729
**C**	2.30808925	0.48025790	0.03544907
**C**	1.96156174	-0.87343474	-0.12075457
**H**	1.78967956	-1.30135258	-1.10015631
**C**	-2.99961802	0.63755102	1.25851805
**H**	-2.63266994	1.66399069	1.18540702

**H**	-2.77197605	0.30309743	2.27669864
**C**	-4.51249203	0.59674441	1.02964731
**H**	-5.03880407	1.21817175	1.75910198
**H**	-4.77202660	0.96455799	0.03180904
**H**	-4.90328603	-0.42182442	1.11804680
**C**	2.57812067	1.29286830	-1.24062349
**H**	1.99653254	0.85939611	-2.06148028
**H**	3.63438845	1.18092637	-1.52055917
**C**	2.24253343	2.78517501	-1.12811788
**H**	2.85167159	3.28063051	-0.36751485
**H**	2.42887803	3.29466479	-2.07729245
**H**	1.19233479	2.93145539	-0.86340714
**H**	2.13986334	-1.59102861	0.66943337
**C**	3.13227362	0.85962791	1.26910233
**H**	4.20639643	0.76137223	1.07369659
**H**	2.94599785	1.89191150	1.57324733
**H**	2.88389012	0.22114318	2.12041388

TS37

Cartesian Coordinates (Å)			
**At.**	**X**	**Y**	**Z**
**H**	1.07550365	0.87576037	0.39595636
**O**	-0.01694200	-1.14500162	0.23408282
**O**	-0.24732071	1.02936164	0.68940345
**C**	-2.23433497	-0.25405680	0.26434588
**H**	-2.47359268	0.02408398	-0.77080061
**H**	-2.48429325	-1.31293615	0.37070969
**C**	-0.72783954	-0.11544479	0.41402577
**C**	2.27867246	0.58324191	0.05574542
**C**	1.97550952	-0.78433130	-0.06811781
**H**	1.82586884	-1.24540711	-1.03608858
**C**	-3.04132031	0.61941208	1.23199295

**H**	-2.69948389	1.65296344	1.13984283
**H**	-2.81981326	0.31471656	2.26080905
**C**	-4.54928397	0.53451927	0.98346619
**H**	-5.10161652	1.15859693	1.69111256
**H**	-4.80381749	0.87278814	-0.02605789
**H**	-4.91533116	-0.49149811	1.08993352
**C**	2.51139309	1.37116200	-1.24490541
**H**	1.88720612	0.92999477	-2.02949885
**H**	3.54884505	1.24260263	-1.57788741
**C**	2.19579322	2.86821650	-1.14315211
**H**	2.84724434	3.37229894	-0.42408181
**H**	2.33759388	3.35844346	-2.11001324
**H**	1.16183491	3.03036173	-0.82813967
**H**	2.16792275	-1.47431165	0.74338084
**C**	3.07182427	1.01254075	1.30065170
**H**	2.84641606	2.05778421	1.53292834
**H**	2.70962868	0.43590728	2.15829762
**C**	4.59181987	0.83857851	1.17250110
**H**	4.99934036	1.43400993	0.35113635
**H**	5.09863497	1.15383198	2.08892781
**H**	4.85593898	-0.20718775	0.98649382

TS38

Cartesian Coordinates (Å)

At.	X	Y	Z
**H**	1.15503317	0.64081808	0.42647312
**O**	-0.07761366	-1.29178483	0.11368534
**O**	-0.12405496	0.85729168	0.72523986
**C**	-2.21285567	-0.22766247	0.24538796
**H**	-2.44186086	0.15125021	-0.75957057
**H**	-2.54709608	-1.26800968	0.27333104
**C**	-0.69929800	-0.22421846	0.37672441

**C**	2.31635672	0.24969358	-0.02516705
**C**	1.92500533	-1.09010503	-0.15869686
**H**	1.75440441	-1.53554063	-1.13140504
**C**	-2.93234146	0.62769153	1.29516037
**H**	-2.51318529	1.63633295	1.27389492
**H**	-2.71760494	0.22811526	2.29241884
**C**	-4.44634037	0.67696525	1.07569988
**H**	-4.93604559	1.28468025	1.84123502
**H**	-4.69136814	1.11056213	0.10091065
**H**	-4.88881182	-0.32343542	1.11289639
**C**	2.62272934	1.09682377	-1.26374560
**H**	1.89475165	0.81879300	-2.03753982
**H**	2.07238473	-1.79647663	0.64695346
**H**	2.93472132	0.46386131	0.85047091
**C**	2.44494098	2.59336795	-0.96922907
**H**	3.13889376	2.91950139	-0.18676728
**H**	2.64499916	3.19567164	-1.85989643
**H**	1.43015102	2.81129859	-0.62867409
**C**	4.02989429	0.80752305	-1.81221813
**H**	4.79372709	1.05497600	-1.06741277
**H**	4.14837085	-0.24881749	-2.07080162
**H**	4.23511364	1.39983421	-2.70935813

TS39

Cartesian Coordinates (Å)			
**At.**	**X**	**Y**	**Z**
**H**	1.14374677	0.77228011	0.38129844
**O**	-0.00637581	-1.21980434	0.12821552
**O**	-0.13903402	0.93947100	0.68942369
**C**	-2.18327947	-0.24045650	0.24768639
**H**	-2.43625240	0.13957098	-0.75100189
**H**	-2.47323420	-1.29406587	0.26883185

**C**	-0.67053097	-0.17264454	0.36946668
**C**	2.31756761	0.41699625	-0.07262265
**C**	1.98274412	-0.94169815	-0.16193842
**H**	1.82818768	-1.42555793	-1.11865666
**C**	-2.92888420	0.57446390	1.31177314
**H**	-2.55064751	1.59930106	1.29747138
**H**	-2.69069084	0.17348547	2.30316725
**C**	-4.44523251	0.56506169	1.10386902
**H**	-4.95301851	1.14507924	1.87905504
**H**	-4.71476669	0.99806962	0.13531602
**H**	-4.84693193	-0.45261462	1.13410218
**C**	2.56652060	1.24009566	-1.33530888
**H**	1.86096962	0.92143182	-2.11137074
**H**	2.16651097	-1.61383925	0.66526635
**H**	2.93146492	0.68190697	0.79113065
**C**	2.42079407	2.74783507	-1.10363064
**H**	3.12487097	3.09805813	-0.34235696
**H**	2.61920650	3.30924242	-2.02042100
**H**	1.41242709	2.99610092	-0.76279583
**H**	3.56886777	1.03023204	-1.72896987

TS40

Cartesian Coordinates (Å)			
**At.**	**X**	**Y**	**Z**
**H**	1.22491772	0.67981993	0.39790556
**O**	-0.00205051	-1.26340099	0.12686271
**O**	-0.05187967	0.89485932	0.70376828
**C**	-2.13769902	-0.19547316	0.22265953
**H**	-2.35739762	0.17254669	-0.78841911
**H**	-2.47331199	-1.23508742	0.25943755
**C**	-0.62531188	-0.19136714	0.36766232
**C**	2.38478005	0.28341448	-0.05725822

**C**	1.99818276	-1.06098198	-0.15420504
**H**	1.82828271	-1.53433166	-1.11351273
**C**	-2.86563841	0.67262035	1.25604090
**H**	-2.44333011	1.67981152	1.22842906
**H**	-2.66230380	0.28286449	2.25955962
**C**	-4.37714709	0.72382440	1.02045040
**H**	-4.87294728	1.34124312	1.77421219
**H**	-4.61077489	1.14749321	0.03851469
**H**	-4.82297530	-0.27483131	1.06388016
**C**	2.66901894	1.10180661	-1.31484113
**H**	1.96064102	0.80881879	-2.09984572
**H**	2.15279136	-1.74312008	0.67078357
**H**	3.00618327	0.51895189	0.80952223
**C**	2.56960585	2.61600357	-1.08807359
**H**	3.26033898	2.90383422	-0.28617144
**H**	1.56461116	2.85759285	-0.72647795
**H**	3.66883407	0.86423895	-1.70229292
**C**	2.88044151	3.43194808	-2.34517761
**H**	2.18350642	3.19283647	-3.15469644
**H**	2.80618518	4.50524542	-2.15132900
**H**	3.89244698	3.22982127	-2.71038839

TS41

Cartesian Coordinates (Å)			
**At.**	**X**	**Y**	**Z**
**H**	1.29728653	0.63405925	0.38541263
**O**	-0.00197275	-1.26016345	0.10339024
**O**	0.02685031	0.89811642	0.68176228
**C**	-2.09550256	-0.10997334	0.18312846
**H**	-2.29315941	0.28120205	-0.82377099
**H**	-2.47077269	-1.13637547	0.20271478
**C**	-0.58529394	-0.16496051	0.33991257

**C**	2.44395815	0.19396687	-0.06160390
**C**	2.00737328	-1.13485485	-0.16068677
**H**	1.82709880	-1.60250299	-1.12089715
**C**	-2.79746718	0.77073252	1.22400661
**H**	-2.33604043	1.76096402	1.21428935
**H**	-2.61742128	0.35867937	2.22308169
**C**	-4.30391365	0.88433763	0.97816223
**H**	-4.78124094	1.50946295	1.73748264
**H**	-4.51304807	1.33124888	0.00101889
**H**	-4.78859188	-0.09668853	1.00329496
**C**	2.76758124	1.00001467	-1.31784338
**H**	2.05326942	0.73314526	-2.10661449
**H**	2.12897654	-1.82137960	0.66620159
**H**	3.06760994	0.40658235	0.80946228
**C**	2.72460618	2.51664172	-1.09156754
**H**	3.42272128	2.78042910	-0.28632707
**H**	1.72722942	2.79806135	-0.73457351
**H**	3.75977424	0.72312735	-1.69829294
**C**	3.07061802	3.32940090	-2.34354416
**H**	2.37286094	3.06654448	-3.14809370
**H**	4.06710929	3.04039744	-2.69970043
**C**	3.03140192	4.84234201	-2.11048996
**H**	3.74557699	5.14171690	-1.33702419
**H**	3.27845577	5.39395057	-3.02168536
**H**	2.03806777	5.16577773	-1.78460670

TS42

Cartesian Coordinates (Å)			
**At.**	**X**	**Y**	**Z**
**H**	0.88286044	1.03540288	0.10183310
**O**	-0.04205964	-1.08787331	0.02537786
**O**	-0.38950649	1.10455677	0.32093280

**C**	-2.29492813	-0.34991881	0.31159218
**H**	-2.71777758	-0.09261781	-0.66822086
**H**	-2.44299168	-1.42330064	0.45238182
**C**	-0.80129233	-0.09619358	0.21813792
**C**	2.14250911	0.68667390	-0.16275260
**C**	1.89146860	-0.69052615	-0.25511443
**H**	1.80728663	-1.18140067	-1.21584116
**C**	-2.99383475	0.46939721	1.40391607
**H**	-2.74955092	1.52597247	1.26321966
**H**	-2.58776399	0.18700063	2.38276529
**C**	-4.51428739	0.27930900	1.41032775
**H**	-4.91727545	0.56851463	0.43202470
**H**	-4.74906262	-0.78490269	1.53336604
**H**	2.10784854	-1.35414019	0.57168731
**H**	2.68007060	1.03588395	0.71527201
**C**	-5.21283057	1.08595565	2.50835337
**H**	-5.02112792	2.15713618	2.39332328
**H**	-6.29571583	0.93705978	2.48508228
**H**	-4.85830627	0.79195658	3.50112698
**H**	2.36426485	1.21605465	-1.08579002

TS43

Cartesian Coordinates (Å)			
**At.**	**X**	**Y**	**Z**
**H**	1.04489367	0.83941796	0.31121456
**O**	-0.02510939	-1.19377744	0.06849071
**O**	-0.23948487	0.95963527	0.63304480
**C**	-2.23862195	-0.33378767	0.32802932
**H**	-2.57413549	0.02513709	-0.65389607
**H**	-2.46691228	-1.40169821	0.37030500
**C**	-0.72716193	-0.18139591	0.35124929
**C**	2.23067468	0.52864245	-0.13937536

**C**	1.94214806	-0.84054932	-0.24087881
**H**	1.80098132	-1.31964590	-1.20206494
**C**	-2.95801707	0.44631642	1.43467275
**H**	-2.63164553	1.48945869	1.40017205
**H**	-2.64485004	0.05904018	2.41175598
**C**	-4.48436569	0.37033640	1.32307308
**H**	-4.79421641	0.76551879	0.34783011
**H**	-4.80038675	-0.67979447	1.33965403
**H**	2.15675125	-1.51414842	0.57768192
**H**	2.83387486	0.80487603	0.72681306
**C**	-5.20430693	1.13703717	2.43585312
**H**	-4.93094211	2.19659220	2.42525478
**H**	-6.29034975	1.07217292	2.32732059
**H**	-4.94459584	0.73924541	3.42179164
**C**	2.44698083	1.36655332	-1.39343193
**H**	1.76530864	1.05862148	-2.19159762
**H**	2.25838155	2.42481339	-1.19656083
**H**	3.46910500	1.27938290	-1.77640027

TS44

	Cartesian Coordinates (Å)		
**At.**	**X**	**Y**	**Z**
**H**	1.16569724	0.73699993	0.37205806
**O**	0.01281849	-1.24818610	0.07625003
**O**	-0.10827410	0.88876067	0.71967606
**C**	-2.16177572	-0.28036532	0.29342828
**H**	-2.44445280	0.15346479	-0.67479851
**H**	-2.44779999	-1.33471325	0.26570258
**C**	-0.64636164	-0.21261938	0.37437645
**C**	2.32955909	0.39913900	-0.12052422
**C**	1.99368304	-0.95591103	-0.25137945
**H**	1.81710340	-1.40467589	-1.22131323

**C**	-2.87571642	0.47251145	1.42259813
**H**	-2.49537942	1.49717718	1.45902952
**H**	-2.61447791	0.01376321	2.38387727
**C**	-4.39957326	0.48415311	1.26232788
**H**	-4.65727656	0.94983769	0.30315417
**H**	-4.76930695	-0.54701521	1.20852103
**H**	2.19779738	-1.65763887	0.54595377
**H**	2.96254973	0.63352749	0.73824529
**C**	-5.11457811	1.22363585	2.39653610
**H**	-4.78723456	2.26616178	2.45562369
**H**	-6.19848489	1.22253080	2.25296025
**H**	-4.90723932	0.75745963	3.36475934
**C**	2.55000747	1.26748917	-1.35800710
**H**	1.82767452	0.97594174	-2.12928522
**H**	3.54354219	1.07341058	-1.78102705
**C**	2.40849640	2.76570362	-1.06901769
**H**	3.12930201	3.08935840	-0.31165037
**H**	2.58580034	3.36004650	-1.96923277
**H**	1.40789885	3.00005386	-0.69684155

TS45

	Cartesian Coordinates (Å)		
**At.**	**X**	**Y**	**Z**
**H**	1.29294592	0.40551290	0.38218984
**O**	0.04533154	-1.47994374	-0.10695398
**O**	0.06065716	0.53131935	0.86886398
**C**	-2.05649792	-0.57991105	0.56129509
**H**	-2.45156898	-0.77798889	-0.44153711
**H**	-2.27371961	-1.48318148	1.14330733
**C**	-0.54274330	-0.50024944	0.43211868
**C**	2.39673786	0.12850164	-0.26402999
**C**	1.99907277	-1.17827717	-0.57902142

**H**	1.71420738	-1.45662081	-1.58620368
**C**	-2.72184524	0.64680196	1.18707827
**H**	-2.48831302	1.53181988	0.58621789
**H**	-2.28325101	0.83268515	2.17265115
**C**	-4.24177250	0.49674624	1.31642160
**H**	-4.67502125	0.30906037	0.32624956
**H**	-4.46931621	-0.39073682	1.91965523
**H**	2.24793669	-2.00632246	0.07075308
**H**	3.11290044	0.20408504	0.55705343
**C**	-4.91268517	1.72294208	1.94164875
**H**	-4.73272581	2.62036878	1.34168796
**H**	-5.99478549	1.58879822	2.02287634
**H**	-4.52464590	1.91540264	2.94664511
**C**	2.53531122	1.18275472	-1.36025616
**H**	1.73234019	1.04231928	-2.09462697
**H**	3.47713791	1.03888632	-1.90656976
**C**	2.48345357	2.62071108	-0.82780660
**H**	3.27165569	2.75269274	-0.07658266
**H**	1.53396714	2.77398698	-0.30422770
**C**	2.64667164	3.67288723	-1.92731378
**H**	1.85184470	3.59006926	-2.67534264
**H**	2.61070659	4.68570609	-1.51748256
**H**	3.60301227	3.55917605	-2.44775815

TS46

Cartesian Coordinates (Å)			
**At.**	**X**	**Y**	**Z**
**H**	1.05645993	0.84089835	0.31614749
**O**	0.03809897	-1.21894846	0.07520469
**O**	-0.21534126	0.91461491	0.69521626
**C**	-2.18856594	-0.42527396	0.41878698
**H**	-2.55947712	-0.07057052	-0.55191868

**H**	-2.38676557	-1.49926607	0.45947325
**C**	-0.68159300	-0.23254327	0.40200617
**C**	2.22940128	0.57616958	-0.19396709
**C**	1.97887332	-0.79933270	-0.30977713
**H**	1.81082440	-1.26519949	-1.27314685
**C**	-2.89970356	0.32829799	1.54890433
**H**	-2.60155902	1.37969331	1.51304766
**H**	-2.55056616	-0.05681497	2.51442041
**C**	-4.42554591	0.21156082	1.47466664
**H**	-4.77230774	0.60493243	0.50996781
**H**	-4.71477836	-0.84735978	1.49200046
**H**	2.24937200	-1.48046838	0.48564371
**H**	2.86196164	0.85519661	0.65012716
**C**	-5.14771131	0.94924239	2.60774225
**H**	-4.85453915	2.00576062	2.59272834
**H**	-4.80498189	0.55304902	3.57119864
**C**	-6.67283797	0.84051951	2.52503632
**H**	-6.99830450	-0.20351368	2.57144290
**H**	-7.15681810	1.37549905	3.34669483
**H**	-7.04919020	1.26182591	1.58753849
**C**	2.36355936	1.44358503	-1.43937316
**H**	1.65677177	1.12905663	-2.21274049
**H**	2.15150079	2.49153757	-1.21340231
**H**	3.36975964	1.39585234	-1.86866748

TS47

Cartesian Coordinates (Å)			
**At.**	**X**	**Y**	**Z**
**H**	2.18561649	0.15585721	0.65065193
**O**	1.22316035	-1.79044144	-0.14690699
**O**	0.86372204	0.28790762	0.59098616
**C**	-0.91729658	-0.81957201	-0.58023551

**H**	-0.86524221	-0.33106483	-1.56226909
**H**	-1.17034269	-1.86727324	-0.76086446
**C**	0.48564895	-0.77541239	0.00087254
**C**	3.45112319	-0.14306995	0.51556654
**C**	3.20139530	-1.48255673	0.18436313
**H**	3.34394650	-1.85341525	-0.82333674
**C**	-1.97135180	-0.12145826	0.28736390
**H**	-1.63091770	0.89437133	0.50606543
**H**	-2.04134674	-0.63614198	1.25306884
**C**	-3.35421837	-0.08219995	-0.37096905
**H**	-3.28219482	0.43990782	-1.33421140
**H**	-3.67983911	-1.10447298	-0.60367895
**H**	3.16233985	-2.24513231	0.95036658
**H**	3.78362959	0.02062032	1.54309142
**C**	-4.41990003	0.60106511	0.49336431
**H**	-4.09211108	1.62064937	0.72868375
**H**	-4.49451780	0.07716001	1.45388222
**C**	-5.79890471	0.64771419	-0.17053457
**H**	-6.16794709	-0.35967459	-0.38742880
**H**	-6.53504052	1.13943723	0.47122956
**H**	-5.76408850	1.19691113	-1.11668766
**C**	4.02206295	0.82050184	-0.52327406
**H**	3.57985331	0.59206532	-1.49995310
**H**	5.10184615	0.65913445	-0.63169644
**C**	3.76348737	2.29127478	-0.17941541
**H**	4.20778814	2.55286139	0.78603711
**H**	4.19594564	2.95491638	-0.93282228
**H**	2.69169346	2.49653085	-0.11830904

TS48

Cartesian Coordinates (Å)			
**At.**	**X**	**Y**	**Z**

**H**	1.27880962	0.60046382	0.40772251
**O**	0.13097877	-1.38686261	0.11009585
**O**	0.01463527	0.73626468	0.79731040
**C**	-2.04296776	-0.43597155	0.39090713
**H**	-2.34291744	-0.03515745	-0.58644315
**H**	-2.32387754	-1.49216355	0.39793129
**C**	-0.52637059	-0.36075267	0.44284833
**C**	2.42766151	0.28350977	-0.13153827
**C**	2.09884057	-1.07207028	-0.27470031
**H**	1.89466646	-1.50641231	-1.24572401
**C**	-2.74433857	0.34651190	1.50728178
**H**	-2.36960614	1.37374596	1.50693747
**H**	-2.46467884	-0.08110975	2.47748169
**C**	-4.27024455	0.34322335	1.36931905
**H**	-4.54749775	0.77755397	0.39969394
**H**	-4.63578424	-0.69186654	1.35352300
**H**	2.33384296	-1.78481248	0.50415531
**H**	3.08728687	0.50742120	0.70970684
**C**	-4.98350006	1.11188907	2.48739065
**H**	-4.61591339	2.14485189	2.50436752
**H**	-4.70880172	0.67632404	3.45563557
**C**	-6.50793675	1.11382910	2.34315205
**H**	-6.90804947	0.09510192	2.35711448
**H**	-6.98555168	1.66875042	3.15525349
**H**	-6.81551292	1.57593101	1.39979584
**C**	2.59991480	1.17409785	-1.36006975
**H**	1.85953544	0.88644203	-2.11701749
**H**	3.58432111	1.00280558	-1.81595841
**C**	2.44775725	2.66924043	-1.05107389
**H**	3.17129788	2.94987515	-0.27599712
**H**	1.45619714	2.84587995	-0.62120954

**C**	2.64799709	3.55859045	-2.28058741
**H**	1.91578839	3.32647925	-3.06046522
**H**	2.53836883	4.61666949	-2.02855620
**H**	3.64464721	3.42172911	-2.71228265

TS49

Cartesian Coordinates (Å)			
**At.**	**X**	**Y**	**Z**
**H**	1.36452185	0.50399471	0.37857522
**O**	0.13836264	-1.43370673	0.06231661
**O**	0.10393023	0.68845090	0.76175323
**C**	-1.99705749	-0.39897568	0.34330856
**H**	-2.27872438	0.02570612	-0.62937006
**H**	-2.31962844	-1.44318788	0.33695947
**C**	-0.47878665	-0.38430433	0.39924771
**C**	2.50322187	0.14336702	-0.15394200
**C**	2.12085089	-1.19693867	-0.30699735
**H**	1.90604983	-1.61747439	-1.28179573
**C**	-2.66916396	0.39706846	1.46818742
**H**	-2.25419999	1.40865177	1.48074777
**H**	-2.40838418	-0.05293684	2.43352491
**C**	-4.19377051	0.45564280	1.32811003
**H**	-4.45187894	0.91214603	0.36339495
**H**	-4.59977637	-0.56397522	1.29931157
**H**	2.32072383	-1.92273772	0.46962226
**H**	3.16500526	0.33613132	0.69328139
**C**	-4.87826679	1.23838491	2.45437507
**H**	-4.47038505	2.25581831	2.48424122
**H**	-4.62266275	0.78085279	3.41771347
**C**	-6.40119900	1.30195772	2.30811407
**H**	-6.84102686	0.29963790	2.30930014
**H**	-6.85814000	1.86547369	3.12617365

**H**	-6.68867852	1.78701717	1.36995611
**C**	2.72013164	1.03276624	-1.37654539
**H**	1.97469696	0.77756979	-2.13997902
**H**	3.70037804	0.82422974	-1.82544780
**C**	2.62421623	2.53066503	-1.05977456
**H**	3.35398628	2.78087381	-0.27861887
**H**	1.63701649	2.74505069	-0.63442186
**C**	2.86461621	3.42769828	-2.27854384
**H**	2.13518741	3.17844168	-3.05897022
**H**	3.85129795	3.20554177	-2.70337757
**C**	2.77319508	4.92171061	-1.95540446
**H**	2.94511776	5.53454703	-2.84449442
**H**	1.78650153	5.17998617	-1.55883535
**H**	3.51572054	5.20985838	-1.20469604

TS50

Cartesian Coordinates (Å)			
**At.**	**X**	**Y**	**Z**
**H**	0.86962058	1.06215210	0.06391207
**O**	0.00670343	-1.08800138	0.02739006
**O**	-0.39618315	1.09466547	0.32448439
**C**	-2.25774354	-0.41521096	0.37506449
**H**	-2.71395706	-0.18335771	-0.59610195
**H**	-2.36921074	-1.49079034	0.53193222
**C**	-0.77549238	-0.11805123	0.23817044
**C**	2.12958206	0.74961059	-0.24201506
**C**	1.91707977	-0.63501601	-0.31869671
**H**	1.81604790	-1.13429401	-1.27345767
**C**	-2.95238510	0.39601136	1.47623414
**H**	-2.74450913	1.45745904	1.31593642
**H**	-2.51078970	0.13855345	2.44639763
**C**	-4.46508872	0.15823405	1.52549524

**H**	-4.90485417	0.42203867	0.55468694
**H**	-4.66456725	-0.91177442	1.66842136
**H**	2.18056560	-1.28660854	0.50398856
**H**	2.68566458	1.12010790	0.61552263
**C**	-5.16953953	0.95378839	2.63022809
**H**	-4.96699114	2.02207273	2.48854572
**H**	-4.73218710	0.68801353	3.60015380
**C**	-6.68242397	0.72125908	2.67210172
**H**	-6.91602634	-0.33374486	2.84656971
**H**	-7.15381207	1.30155219	3.46992816
**H**	-7.15360494	1.01160445	1.72782205
**H**	2.30409781	1.27972696	-1.17471245

TS51

Cartesian Coordinates (Å)			
**At.**	**X**	**Y**	**Z**
**H**	0.87587939	1.06995107	0.03752563
**O**	0.02317310	-1.08411465	-0.00799664
**O**	-0.38646091	1.09356236	0.31537783
**C**	-2.23937723	-0.42613492	0.37902401
**H**	-2.71192568	-0.18270152	-0.58143088
**H**	-2.34378105	-1.50424287	0.52283840
**C**	-0.76082156	-0.12026457	0.22317284
**C**	2.13320185	0.76670521	-0.28785426
**C**	1.92647091	-0.61818748	-0.37503177
**H**	1.81526077	-1.10881229	-1.33314298
**C**	-2.91974736	0.36688926	1.50222722
**H**	-2.71789868	1.43120991	1.35383942
**H**	-2.46240009	0.09723576	2.46170175
**C**	-4.43090343	0.12301813	1.57115821
**H**	-4.88538307	0.39930972	0.61079210
**H**	-4.62372844	-0.94972049	1.70117452

**H**	2.20411113	-1.27628616	0.43776369
**H**	2.69875394	1.13178658	0.56580287
**C**	-5.11977734	0.90030798	2.69816140
**H**	-4.92331206	1.97252663	2.57011051
**H**	-4.66765336	0.62202938	3.65896151
**C**	-6.63347217	0.66913101	2.76828296
**H**	-6.82912332	-0.40257822	2.89517031
**H**	-7.08556963	0.94851829	1.80887640
**H**	2.29281843	1.30654566	-1.21763941
**C**	-7.31295787	1.44843949	3.89778117
**H**	-7.16447362	2.52631479	3.77927029
**H**	-8.39022362	1.26243434	3.92026246
**H**	-6.90668491	1.16512844	4.87382339

TS52

Cartesian Coordinates (Å)			
**At.**	**X**	**Y**	**Z**
**H**	0.86833690	1.09898807	0.00630044
**O**	0.05723194	-1.07135813	-0.02602981
**O**	-0.38410163	1.09546163	0.32635318
**C**	-2.20792755	-0.45861395	0.41474329
**H**	-2.70072623	-0.23400820	-0.54020149
**H**	-2.28743112	-1.53752821	0.56841812
**C**	-0.73873144	-0.12403390	0.23111240
**C**	2.11897561	0.82322529	-0.36610472
**C**	1.93778249	-0.56524644	-0.45290365
**H**	1.80452449	-1.05434051	-1.40898856
**C**	-2.88715251	0.32932526	1.54196111
**H**	-2.70880440	1.39628819	1.38283707
**H**	-2.41031429	0.07642008	2.49648418
**C**	-4.39198565	0.05606942	1.63515102
**H**	-4.86586040	0.31520908	0.67940303

**H**	-4.56154844	-1.01913689	1.77661505
**H**	2.25585403	-1.22060846	0.34717065
**H**	2.70650844	1.19616676	0.46913890
**C**	-5.07978646	0.82921219	2.76592612
**H**	-4.90531899	1.90361617	2.62592155
**H**	-4.60795687	0.56736381	3.72170802
**C**	-6.58727782	0.56882042	2.85873773
**H**	-6.76185187	-0.50608593	2.99803586
**H**	-7.05994752	0.83107152	1.90319770
**H**	2.23476267	1.37038111	-1.29808567
**C**	-7.27643994	1.34112662	3.98939856
**H**	-7.10236238	2.41498359	3.85025338
**H**	-6.80504772	1.07889112	4.94434455
**C**	-8.78214875	1.07551576	4.07431218
**H**	-8.98743509	0.01455631	4.24837779
**H**	-9.24278015	1.64041648	4.88938764
**H**	-9.28704717	1.36085275	3.14602823

TS53

Cartesian Coordinates (Å)

At.	X	Y	Z
**H**	0.87119759	1.10845456	-0.02577202
**O**	0.06605221	-1.06397920	-0.06050360
**O**	-0.37968444	1.10059241	0.30031801
**C**	-2.19760273	-0.45927090	0.39961735
**H**	-2.70092877	-0.22387125	-0.54716670
**H**	-2.27394784	-1.53996369	0.54183974
**C**	-0.73100878	-0.11985619	0.20490137
**C**	2.12102974	0.83727014	-0.40402011
**C**	1.94305709	-0.55139965	-0.49462497
**H**	1.80671368	-1.03760859	-1.45174495
**C**	-2.86554772	0.31469943	1.54332042

**H**	-2.69006745	1.38364326	1.39456345
**H**	-2.37808962	0.05122030	2.48960193
**C**	-4.36900447	0.03863841	1.64969061
**H**	-4.85349118	0.30854933	0.70227534
**H**	-4.53591252	-1.03837499	1.78021532
**H**	2.26662649	-1.20861557	0.30172518
**H**	2.71115000	1.20900822	0.42992966
**C**	-5.04522218	0.79761706	2.79697603
**H**	-4.87275890	1.87372376	2.66818680
**H**	-4.56323004	0.52452649	3.74447803
**C**	-6.55174582	0.53509919	2.90233663
**H**	-6.72336547	-0.54158645	3.03003831
**H**	-7.03346202	0.80855087	1.95463481
**H**	2.23144724	1.38774912	-1.33469517
**C**	-7.22849963	1.29356341	4.04949685
**H**	-7.05701690	2.37043734	3.92262388
**H**	-6.74786452	1.02025704	4.99793132
**C**	-8.73538940	1.03290487	4.15553317
**H**	-8.90696437	-0.04288069	4.28289799
**H**	-9.21591027	1.30640407	3.20829481
**C**	-9.40284647	1.79478942	5.30402948
**H**	-9.27893335	2.87587945	5.18608830
**H**	10.47536563	1.58722853	5.35204057
**H**	-8.96742343	1.51660189	6.26894728

TS54

Cartesian Coordinates (Å)			
**At.**	**X**	**Y**	**Z**
**H**	0.85341232	1.14402184	-0.08413142
**O**	0.09242355	-1.04454492	-0.07071819
**O**	-0.39310916	1.11672710	0.25713287
**C**	-2.17762763	-0.47712678	0.40590875

**H**	-2.69800305	-0.26303087	-0.53668688
**H**	-2.23133783	-1.55715612	0.56268452
**C**	-0.72058757	-0.11208653	0.18744280
**C**	2.10398984	0.89128355	-0.47259616
**C**	1.95336220	-0.50211766	-0.53623154
**H**	1.81591121	-1.00823207	-1.48281878
**C**	-2.84456805	0.29861630	1.54907237
**H**	-2.69059284	1.36864151	1.38500419
**H**	-2.33988057	0.05561850	2.49181106
**C**	-4.34121862	-0.00304708	1.67915314
**H**	-4.84322898	0.24722425	0.73547088
**H**	-4.48695980	-1.08128256	1.82414820
**H**	2.29953520	-1.13813564	0.26778889
**H**	2.69614938	1.28989497	0.34737731
**C**	-5.01518013	0.75708478	2.82701425
**H**	-4.86303747	1.83447495	2.68401174
**H**	-4.51590340	0.50300117	3.77086366
**C**	-6.51540663	0.47013985	2.95578518
**H**	-6.66687421	-0.60782122	3.09729017
**H**	-7.01450973	0.72502498	2.01194257
**H**	2.19226257	1.42691101	-1.41423901
**C**	-7.18928413	1.22977627	4.10415461
**H**	-7.03645910	2.30756194	3.96308921
**H**	-6.69092123	0.97415148	5.04821112
**C**	-8.68988359	0.94530563	4.23242088
**H**	-8.84374768	-0.13244633	4.37416293
**H**	-9.18911128	1.20069413	3.28857598
**C**	-9.36486430	1.70507545	5.38007823
**H**	-9.21180518	2.78185570	5.23845268
**H**	-8.86687766	1.44969909	6.32334965
**C**	10.86364096	1.41481928	5.50022821

**H**	11.04736568	0.35044248	5.67750408
**H**	11.31412676	1.97120294	6.32678136
**H**	11.39494528	1.69278024	4.58451686

TS55

	Cartesian Coordinates (Å)		
**At.**	**X**	**Y**	**Z**
**H**	0.86142566	1.15089597	-0.09935581
**O**	0.09827430	-1.03705527	-0.11133498
**O**	-0.38153910	1.11940142	0.25421303
**C**	-2.16679634	-0.47479872	0.39360857
**H**	-2.69300197	-0.25378288	-0.54417350
**H**	-2.21910494	-1.55612981	0.54182279
**C**	-0.71121854	-0.10778692	0.16847403
**C**	2.10758421	0.90293069	-0.50489779
**C**	1.95494418	-0.48921931	-0.58791959
**H**	1.80718415	-0.98093563	-1.54052051
**C**	-2.82724390	0.29141731	1.54684022
**H**	-2.67438568	1.36279262	1.39072695
**H**	-2.31709299	0.04094064	2.48466110
**C**	-4.32309040	-0.01164139	1.68302064
**H**	-4.83052662	0.24600940	0.74423012
**H**	-4.46780298	-1.09104176	1.82023677
**H**	2.30879895	-1.13751847	0.20283692
**H**	2.70870128	1.28865197	0.31473002
**C**	-4.99075395	0.73915007	2.84070647
**H**	-4.83967767	1.81769561	2.70542265
**H**	-4.48602617	0.47774722	3.77964682
**C**	-5.01518013	0.45074428	2.97572429
**H**	-4.86303747	-0.62836789	3.10952301
**H**	-4.51590340	0.71294326	2.03676951
**H**	-6.51540663	1.45265319	-1.43922044

**C**	-6.66687421	1.20099324	4.13390263
**H**	-7.01450973	2.27991725	4.00052628
**H**	2.19226257	0.93820093	5.07301848
**C**	-7.18928413	0.91459126	4.26835406
**H**	-7.03645910	-0.16443293	4.40172810
**H**	-6.69092123	1.17738305	3.32909416
**C**	-8.68988359	1.66485650	5.42612047
**H**	-8.84374768	2.74414418	5.29351031
**H**	-9.18911128	1.40226411	6.36610384
**C**	-9.36486430	1.37959378	5.56119604
**H**	-9.21180518	0.30144639	5.69433414
**H**	-8.86687766	1.64243942	4.62248427
**C**	10.86364096	2.13341636	6.72018829
**H**	11.04736568	3.21611575	6.59737750
**H**	11.31412676	1.90823704	6.78883898
**H**	11.39494528	1.86514015	7.67745907
